# Differential contribution to gene expression prediction of histone modifications at enhancers or promoters

**DOI:** 10.1371/journal.pcbi.1009368

**Published:** 2021-09-02

**Authors:** Mar González-Ramírez, Cecilia Ballaré, Francesca Mugianesi, Malte Beringer, Alexandra Santanach, Enrique Blanco, Luciano Di Croce

**Affiliations:** 1 Centre for Genomic Regulation (CRG), Barcelona Institute of Science and Technology (BIST), Barcelona, Spain; 2 CNAG-CRG, Centre for Genomic Regulation (CRG), Barcelona Institute of Science and Technology, Barcelona, Spain; 3 Universitat Pompeu Fabra (UPF), Barcelona, Spain; 4 ICREA, Pg. Barcelona, Spain; University of Virginia, UNITED STATES

## Abstract

The ChIP-seq signal of histone modifications at promoters is a good predictor of gene expression in different cellular contexts, but whether this is also true at enhancers is not clear. To address this issue, we develop quantitative models to characterize the relationship of gene expression with histone modifications at enhancers or promoters. We use embryonic stem cells (ESCs), which contain a full spectrum of active and repressed (poised) enhancers, to train predictive models. As many poised enhancers in ESCs switch towards an active state during differentiation, predictive models can also be trained on poised enhancers throughout differentiation and in development. Remarkably, we determine that histone modifications at enhancers, as well as promoters, are predictive of gene expression in ESCs and throughout differentiation and development. Importantly, we demonstrate that their contribution to the predictive models varies depending on their location in enhancers or promoters. Moreover, we use a local regression (LOESS) to normalize sequencing data from different sources, which allows us to apply predictive models trained in a specific cellular context to a different one. We conclude that the relationship between gene expression and histone modifications at enhancers is universal and different from promoters. Our study provides new insight into how histone modifications relate to gene expression based on their location in enhancers or promoters.

## Author summary

Gene expression can be properly predicted by the ChIP-seq signal of histone modifications at promoters, but whether this is also true at enhancers is unclear. In this study we develop predictive models of gene expression that demonstrate the predictive power of histone modifications at enhancers in the context of mouse embryonic stem cells, during differentiation, and in animal development. Moreover, by assessing the contribution of each histone modification, we found that enhancer predictive models and promoter predictive models have different histone modification requirement. Therefore, different histone modifications relate better to enhancer or promoter function(s). Finally, by applying predictive models trained in a specific cellular context to a different one, we concluded that the relationship between gene expression and histone modifications at enhancers is universal.

## Introduction

Appropriate regulation of gene expression is necessary for correct development and homeostasis of organisms. Different classes of regulatory genomic regions are coordinated to establish the appropriate gene transcriptional programs in every cell. These regulatory elements include, among others, promoters and enhancers [1]. Promoters are non-coding DNA fragments located in the surroundings of a transcriptional start site (TSS) that initiate gene transcription, whereas enhancers are distal non-coding DNA fragments that amplify gene expression [1]. DNA is wrapped around histones to form nucleosomes, which are the basic structural unit of chromatin. Post-translational modifications at histones can affect chromatin function by altering its structure, for example by facilitating or preventing the accessibility of transcription factors (TFs) to certain genomic regions [2]. Distinct histone modifications at regulatory elements are associated with gene activation, such as trimethylation of histone H3 at lysine 4 (H3K4me3) [3–5], and acetylation of histone H3 at lysine 27 (H3K27ac) [6], or with gene repression, such as trimethylation of histone H3 at lysine 27 (H3K27me3) [7]. In contrast, monomethylation of histone H3 at lysine 4 (H3K4me1) is a histone modification associated with enhancers [8]. Combinations of histone modifications can have synergistic or antagonist effects on gene regulation. Promoters and enhancers are in fact decorated by a particular combination of different histone modifications according to the transcriptional state of their target gene.

Nowadays, RNA-seq is the main technique to assess gene expression levels, while ChIP-seq experiments allow to map histone modifications genome-wide. Indeed, much effort has been made to understand the quantitative relationship between ChIP-seq levels of histone modifications and gene expression in different cellular contexts [9–15]. However, none of these studies have introduced epigenetic information of enhancers into the modelling for predicting gene expression, but rather have focused only on promoters or gene bodies. Indeed, gene expression has been alternatively modelled using data on chromatin accessibility at promoters in combination to enhancers, together with information about TFs and chromatin remodelers [16]. In this regard, it has been recently shown that including information to the promoter predictive models about chromatin accessibility and the predicted affinity of TF for enhancers can improve the model performance significantly [17]. However, the independent contribution of enhancer and promoter information separately has not been evaluated yet. Although ChIP-seq levels of H3K27ac at enhancers have been modelled with gene expression to (i) obtain predictive models of differential gene expression across tissues and conditions [18], and to (ii) identify enhancer-promoter associations [19], yet to our knowledge, multiple histone modifications exclusively at enhancers have not been used to generate predictive models of gene expression. Therefore, we consider that modelling gene expression from enhancer epigenetic information might help to understand how the contribution to gene expression differs between promoters and enhancers and, more broadly, how enhancers function.

Here, we set out to explore the quantitative relationship between histone modifications and gene expression, focusing on enhancer regions. Our main goal is to decipher which histone modifications correlate with enhancer function. To do so, we asked the following questions: (i) are histone modifications at enhancers predictive of gene expression? (ii) Which histone modifications are more predictive in the enhancer models? (iii) Are the same histone modifications also important for the promoter predictive models? (iv) Is an enhancer predictive model learned in a specific cell type useful to predict gene expression in another one? To address these issues, we developed a novel computational approach based on the combination of chromatin segmentation and linear regression to infer gene expression using ChIP-seq data from histone modifications at enhancers and promoters. To construct proper predictive models, the full spectrum of active and repressed regions is needed. Therefore, we took advantage of mouse embryonic stem cells (ESCs) for which active and repressed regulatory regions can be identified. ESCs contain active enhancers (AEs) and poised (repressed) enhancers (PEs), which respectively coordinate with active promoters (APs) and bivalent (repressed) promoters (BPs) to regulate gene expression [20].

We first performed ChIP-seq experiments of several histone modifications to identify all four types of regulatory regions and to model gene expression in ESCs. Next, as BPs and PEs can either be activated or remain repressed during later stages of differentiation [20], we have applied our framework to predict gene expression in “in vitro” and “in vivo” differentiated cells. Further, we successfully predicted gene expression in a differentiation time point different from the one in which the model was built. To overcome potential pitfalls of using information from different sources (e.g. cell types, labs, etc.), we applied a normalization approach based on a local regression (LOESS) method. LOESS normalization has shown to be useful for normalizing RNA-seq and ChIP-seq data coming from different sources. We found that histone modification levels at enhancers, as well as at promoters, can predict gene expression of their target genes. Remarkably, we determined that BPs and also PEs are good predictors of gene expression at later stages of differentiation and development. Notably, we also observed that histone modifications have different contributions depending on their location at enhancers or promoters. We propose that the relationship of gene expression and histone modifications at PEs is universal, as we have successfully predicted gene expression in a specific cell type using a model previously trained in another one.

## Results

### Identification of promoters and enhancers in ESCs

**Fig 1:**
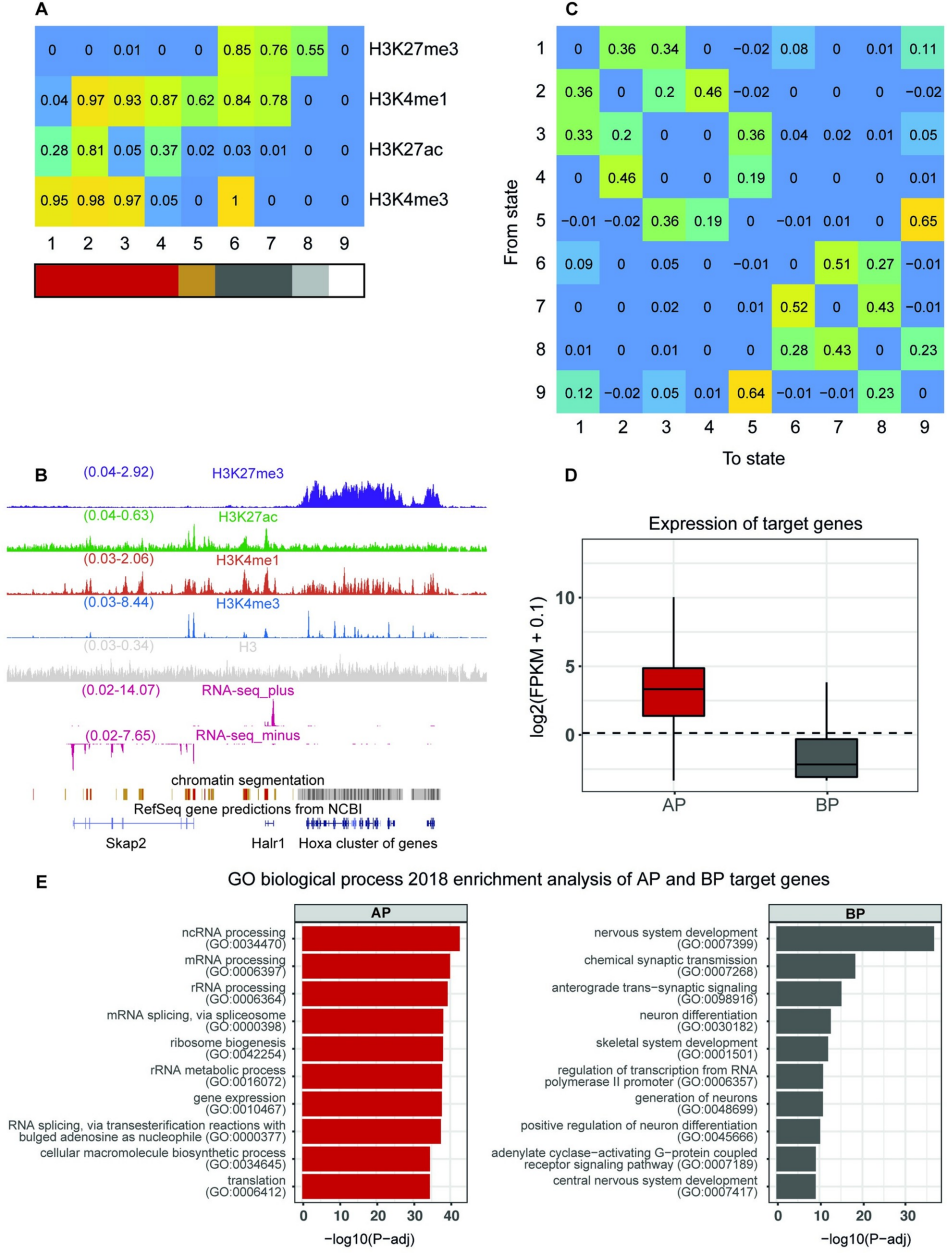
Identification of repressed and active functional regions in ESCs. (A) State definition of the chromatin segmentation model in ESCs. The values represent the probability (from 0 to 1) of finding each histone modification (vertical) in genomic segments of states 1 to 9 (horizontal). The cells of the matrix are colored according to the value of probability they contain inside. Red: states with histone modifications associated to activation (active, 1–4); dark yellow, H3K4me1-only state (Intermediate, 5); grey, states in which H3K27me3 was present (repressed, 6–8); dark grey, poised states, in which H3K27me3 colocalized with H3K4me3 and/or H3K4me1 (states 6 and 7); light grey, H3K27me3-only regions (state 8); and white, unmarked state (9). (B) Example of a genomic region containing two expressed genes (*Skap2* and *Halr1*), which are covered by active states (in red), and a cluster of repressed genes (*HoxA*), which are covered by repressed states (in grey). Active chromatin segments integrate the signal of H3K27ac, H3K4me3, and H3K4me1 and lack H3K27me3. Repressed chromatin segments integrate the signal of H3K27me3, H3K4me3, and H3K4me1 and lack H3K27ac. Expression of *Skap2* and *Halr1*, and silencing of *HoxA* genes, were confirmed by the RNA-seq profiles [23]. Y-axis represents normalized count of reads by total reads. The screenshot was taken from the UCSC Genome Browser [62]. (C) Enrichment of state transitions (e.g., number of observed transitions divided by the number of expected transitions by chance) from the segments of one state (vertical) towards the segments of another state (horizontal) in the linear chromatin. The cells of the matrix are colored according to the value of enrichment they contain inside. (D) Expression of genes associated to active promoters (AP; 10,786 genes) or bivalent promoters (BP; 3,459 genes). The dotted line represents 1 FPKM. (E) Top GO biological process (2018 categories) for each list of genes in D.

We performed ChIP-seq experiments of H3K4me3, H3K27me3, H3K27ac, and H3K4me1 in mouse ESCs to identify the different types of regulatory regions. This set of histone modifications has been previously used to distinguish between AEs, PEs, APs, and BPs in mouse ESCs [21,22]. We next generated a 9-state chromatin segmentation model of ESCs using ChIP-seq data (Fig 1A and 1B). As expected, active states mark transcriptionally active regions, while repressed states denote transcriptionally repressed regions (Fig 1B; see also our previously published RNA-seq data [23]).

To understand the resulting map of states, we calculated the matrix of transition enrichments between all states in the model. The transition value between two different states, *x* and *y*, is defined as the number of times that a segment of state *y* is found after a segment of state *x*, as measured from left to right in the linear genome. The enrichment score is defined as the ratio between the observed number of transitions and the expected number of transitions by chance. Two groups of states (active, 1–4, and repressed, 6–8) clearly emerged from the global picture (Fig 1C) (note that state 5 represents a distinct category; see Discussion). The high enrichment of transitions between states belonging to the same category suggests that they might mark the same functional regulatory regions, rather than be caused by different functional regions separated by the unmarked stated. Visual inspection confirmed that states 1 to 4 marked the same active regulatory regions, revealing that differences in the state definition are due to differences in the shape of the ChIP-seq peaks (S1A Fig). We also observed that the repressed states 6 and 7 decorated poised or bivalent regulatory regions (e.g., marked with H3K27me3, in combination with H3K4me3 and/or H3K4me1), whereas state 8 was generated by the tail-end of broad peaks of H3K27me3 (S1B Fig). Similarly, state 5 was associated with the tail-end of broad peaks of H3K4me1 in active regions, but it also associated with single peaks of H3K4me1 near active regions (S1A Fig).

Based on these results, we decided to merge the contiguous segments of states 1 to 4 as a list of potential active regulatory regions, and the segments of states 6 and 7 as a list of potential poised or bivalent regulatory regions. We reasoned that functional regions should have a minimum length of 600 bp and thus discarded shorter regions, which we considered as background signal. We also discarded those cases in which an active region and a poised region were contiguous, as this was ambiguous. Promoters were defined as those regions that overlapped by at least 1 bp to a region ± 500 bp around a TSS according to RefSeq [24]. Enhancers were defined as regions that were not classified as promoters and overlapped by at least 1 bp with a peak of the enhancer mark p300 [21]. As H3K4me3 can be present in enhancers [25–28], we did not discard enhancers containing H3K4me3, although this histone modification has been traditionally only associated with promoters. In total, we found 9,421 APs, 3,344 BPs, 16,904 AEs, and 2,699 PEs (S1–S4 Tables).

Next, we matched our set of promoters to their target genes, using the same parameters as before (overlap by at least 1 bp to a region ± 500 bp around a TSS according to RefSeq [24]). Using RNA-seq data [23], we confirmed that genes associated with APs are expressed, while genes associated with BPs are not (Fig 1D). Gene ontology (GO) term enrichment analysis performed with Enrichr [29] confirmed that genes with APs are involved in housekeeping roles, while genes with BPs are mostly related to development and differentiation (Fig 1E), as is expected in mouse ESCs. On the other hand, we used available high-throughput chromosome conformation capture (3C) data of Hi-C [30], to link AEs and PEs with target genes. As interacting enhancers and promoters have been shown to match their chromatin state [31], we associated an enhancer with the target gene of a promoter when both enhancer and promoter are in the same category (e.g., both active, or both repressed) and each one overlaps with one of the two sides of the same Hi-C significant interaction (total of 43,892,155 significant Hi-C interactions). We confirmed that PEs were significantly enriched in interactions with BPs over APs (*p* < 2.2e-16 Exact Binomial Test, observed probability: 0.29, expected probability by chance: 0.24), whereas AEs were significantly enriched in interactions with APs over BPs (*p* < 2.2e-16 Exact Binomial Test, observed probability: 0.80, expected probability by chance: 0.76). In total, we found 10,786 genes associated to APs, 3,459 genes to BPs, 10,206 genes to AEs, and 2,526 genes to PEs (S1, S2, S5 and S6 Tables). Likewise, 15,841 AEs and 2,466 PEs were associated to at least one gene, and 8,931 APs and 2,443 BPs, to at least one enhancer (S5 and S6 Tables).

### Development of a predictive model of gene expression using histone modifications at enhancers

After identifying the set of enhancers and promoters in ESCs, we built the gene expression predictive models. We first performed additional ChIP-seq experiments for other histone modifications, in order to have additional variables to predict gene expression. Specifically, we performed ChIP-seq experiments for trimethylation of histone H3 at lysine 36 (H3K36me3), ubiquitination of histone H2B (H2Bub), monomethylation of histone H3 at lysine 27 (H3K27me1), dimethylation of histone H3 at lysine 27 (H3K27me2), trimethylation of histone H4 at lysine 20 (H4K20me3), and dimethylation of histone H3 at lysine 79 (H3K79me2). The input of our predictive models consisted of the ChIP-seq data of these six histone marks as well as the four histone marks previously used to define promoters and enhancers (H3K4me3, H3K4me1, H3K27ac and H3K27me3), together with our previously-published RNA-seq expression data [23].

Initial studies on gene expression prediction revealed that using two to three histone modifications (rather than larger set) are sufficient to accurately predict gene expression, and do not find substantial improvements with the addition of other histone modifications into the models [9–11]. Indeed, follow up publications directly utilize three to four histone modifications [12, 13]. However, our objective is not only to predict gene expression, but also to assess histone modification contribution in enhancer predictive models in comparison to promoter predictive models. Therefore, we used a set of ten histone modifications, each one with different properties (associated to activation, associated to repression, broad marks, sharp marks, etc.). A total of 11,387 protein coding genes previously associated to a promoter (active or bivalent) and at least one enhancer (active or poised) entered the modelling. We divided the set of genes into two subsets: training and test. The Pearson’s correlation coefficient (*r*) between the measured expression in the test subset and the predicted one was used to assess performance.

As one of our aims is to compare enhancer predictive models to promoter predictive models, we needed to build them using the same approach to identify enhancers and promoters so they are comparable. Therefore, we first generated a predictive model for the promoters identified using our approach (named Hi-C–all promoter model) to confirm that histone modifications at these elements are predictive of gene expression. The predictive capacity of promoters has been previously shown in several cell types from different model organisms, including ESCs, where promoters were defined as a pre-set distance from a TSS [9–15]. As a control, we repeated the predictive model learning in a training subset in which expression values were randomized. We obtained an *r*-value of 0.81 for the promoter model, and an *r*-value of –0.07 for the random promoter model (S2A Fig). The low performance of the random promoter model strongly indicated that the high predictive power of the Hi-C–all promoter model was not due to random structures in the data. Importantly, the performance of our promoter model was comparable to previously described predictive models, in which *r*-values around 0.8 were reported [9–15]. Indeed, Karlic and colleagues obtained an *r*-value of 0.77 in CD4+ T-cells in the seminal paper on gene expression prediction from histone modification levels [9]. Coefficients and *p*-values of the predictors for all the predictive models generated in this study can be found in S7 Table.

**Fig 2:**
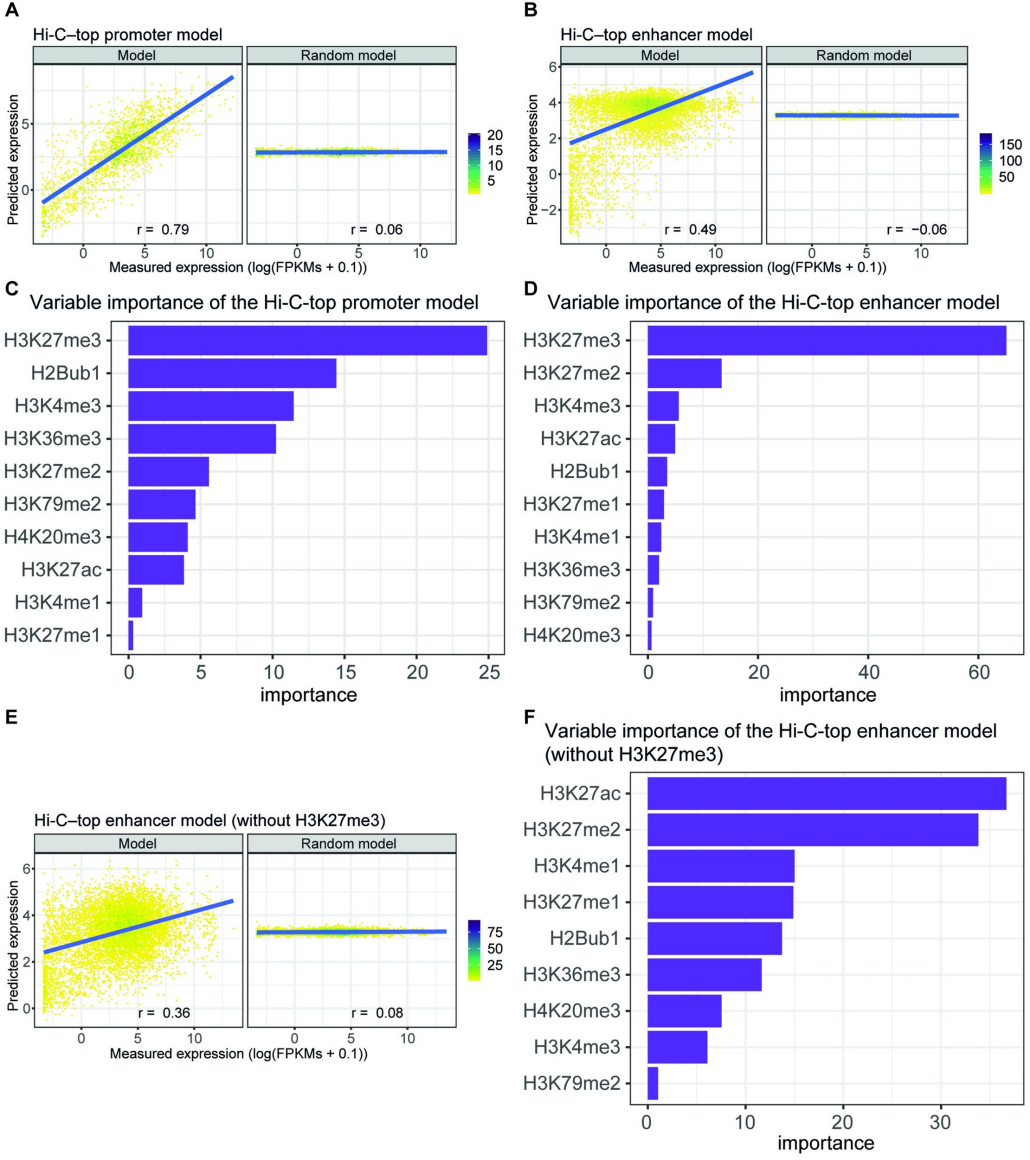
Performance and variable importance of enhancer and promoter Hi-C-top predictive models in ESCs. Predicted expression of the test subset of genes calculated by the models versus their measured expression by RNA-seq. Model performances are represented by the Pearson’s correlation (*r*) between predicted and measured expression values. (A) Left, the model trained on the promoter regions associated to at least one enhancer using the top significant interactions of Hi-C (Hi-C–top promoter model). Right, the performance of the same model after randomizing the expression of the training subset of genes. The color bar represents the density of dots. (B) Left, the model trained on the enhancer regions associated to at least one promoter using the top significant interactions of Hi-C (Hi-C–top enhancer model). Right, the performance of the same model after randomizing the expression of the training subset of genes. The color bar represents the density of dots. (C) Importance of each histone modification used to train the Hi-C–top promoter predictive model. Importance is defined as the contribution of each variable in the linear regression predictive model and corresponds to the absolute value of the t-statistic for each model parameter. (D) As for C, but for the Hi-C–top enhancer predictive model. (E) As for B, but the model is trained without H3K27me3 as predictive variable. (F) As for D, but the model is trained without H3K27me3 as predictive variable.

Next, we trained a second predictive model of gene expression (the Hi-C–all enhancer model) using the levels of histone modifications at the previously-defined enhancers as predictors. We obtained a performance in the test subset of r = 0.38 (Fig 2B). Although the *r*-value of this model is still modest, this is the first time to our knowledge that enhancers have been shown to be predictors of gene expression through their histone modification levels. Critically, when the expression data for learning the model were randomized, the performance was poor (*r* = –0.15, Fig 2B).

We hypothesized that the modest performance of the Hi-C–all enhancer model could be due to some enhancer–promoter associations that were simply regions in close 3D proximity but not functionally linked. To enrich the set of interactions for functional promoter–enhancer loops, we applied a more restrictive threshold on the Hi-C significant interactions (FDR = 0 and ln(*p*-value) ≤ –100). We obtained a total of 5,555,844 interactions (8% of the total interactions). We then recalculated the enhancer-promoter-gene associations and obtained 1,846 PEs associated to 1,382 BPs and to 1,434 target genes, and 11,777 AEs associated to 7,211 APs and to 8,254 target genes (S8 and S9 Tables). We selected the protein-coding genes included in the new associations (a total of 8,639 genes) to recalculate the predictive models (hereon in termed Hi-C–top models) and random models for promoters (Fig 2A) and enhancers (Fig 2B). While the Hi-C–top promoter model performed similarly to the previous model (*r* = 0.79 vs. *r* = 0.81, respectively), the Hi-C–top enhancer model was significantly improved (*r* = 0.49 vs. *r* = 0.38). This result further confirmed that enhancers, as well as promoters, possess a quantitative relationship with gene expression.

We now know that an enhancer preferentially interacts with promoters located in the same topologically associating domain (TAD) rather than those located in neighboring domains [32]. Moreover, TADs have an average size of around 1 Mb. Therefore, assigning genes to enhancers located in a distance lower than 1 Mb might seem appropriate. Indeed, when evaluating a new ESC predictive model that associates enhancers to promoters of the same chromatin state that are closer than 1 Mb (1 Mb model; 11,986 protein-coding genes), we achieved a performance of *r* = 0.34 (S2C Fig). Nonetheless, that performance is lower than the ESC models based on Hi-C data that we have built previously (*r* = 0.38 and *r* = 0.49 for Hi-C–all and Hi-C–top enhancer models, respectively). This suggests that matching genes to regulatory elements by 1 Mb distance leads to some false-positive associations, yet maintaining its predictive capacity.

Finally, from the Hi-C–top interactions we selected those involving a distal enhancer (> 5 Kb from a TSS, a total of 5,235 AEs and 696 PEs) to confirm that the predictive capacity was not exclusive of proximal enhancers. We generated a new distal enhancer model (Hi-C–top_distal; 7,925 protein-coding genes) that properly predicted gene expression with a *r* = 0.41 (S2D Fig).

Enhancers and promoters exhibit similar histone modification patterns. We therefore wondered whether the histone modifications mostly contributing to the prediction of expression were the same ones as well, or whether there were differences in the contributions between the enhancer and the promoter predictive models. To address this, we assessed variable importance in the Hi-C–top model for promoters and enhancers. Notably, H3K27me3—a histone modification associated with transcriptional gene repression—was the prevalent mark in both classes of regulatory elements (Fig 2C and 2D). In contrast to promoters, in which H3K27me3 has a relatively similar importance as other marks (e.g., H2Bub, H3K4me3, and H3K36me3, Fig 2C), H3K27me3 in enhancers represented up to 55% of the total importance (Fig 2D). Therefore, even though promoters and enhancers contribute to predict gene expression mostly through H3K27me3, this contribution seems to be uniquely driven by H3K27me3 in the enhancer predictive model and shared by other histone marks in the promoter predictive model.

Interestingly, H3K27ac—considered the canonical marker of enhancer activation [33]—had little importance in the enhancer model. H3K27me3 and H3K27ac have antagonistic effects and are generally mutually exclusive marks since they occur on the same lysine and are chemically prohibited [34]. Thus, we reasoned that H3K27ac importance was masked by H3K27me3 presence in the predictive model, as both histone modifications are not independent variables. Indeed, when excluding H3K27me3 as a predictive variable, the enhancer model maintains its predictive capacity (*r* = 0.36; Fig 2E), and the most important variable is in fact H3K27ac (Fig 2F). However, in this case, H3K27ac has a relatively similar importance as H3K27me2. Therefore, H3K27me3 seems more predictive than H3K27ac at enhancers in relationship with the rest of the histone modifications.

### LOESS normalization of ChIP-seq and RNA-seq data from heterogeneous sources

We next wanted to determine whether enhancers are predictive of gene expression in other cellular contexts besides ESCs, and whether a predictive model learned in one cell type could predict gene expression in another. As true colocalization of H3K27me3 with H3K4me1 or p300 in the same DNA fragment has been only studied in ESCs, it is still not clear whether PEs exist in other developmental scenarios [20]. To address this issue, we took advantage of the capacity of PEs and BPs to switch into an active state for certain cell types, in a lineage-specific manner during differentiation from ESCs, while remaining inactive in others [20]. We hypothesized that differentiation data could be used to obtain predictive models exclusively from PEs and BPs. We focused on several time points for two cell differentiation mouse models: i) cardiac lineage: mesoderm, cardio precursors, and cardiomyocytes [35]; and ii) neural lineage: neural precursors and cortical neurons [30].

We downloaded RNA-seq and ChIP-seq data of five histone modifications (H3K27me3, H3K4me3, H3K27ac, H3K4me1, and H3K36me3) that were available in the literature for both differentiation models. To remove potential biases (e.g., due to the source of data generation or to a batch effect), we normalized the sequencing samples of the same feature at all the available time points. For this, we applied a normalization based on a local regression (LOESS) that was originally proposed for the pairwise normalization of expression microarrays [36] but generalized for multiple arrays [37]. LOESS normalization is based on a MA methodology, where M is the log_2_ ratio of the intensities of the samples, and A is the log_2_ of the average intensity. It assumes that the intensities of the two samples should be equal, therefore M = 0. Finally, corrections based on a LOESS are applied to obtain a MA plot in where the regression line approximates M = 0.

**Fig 3:**
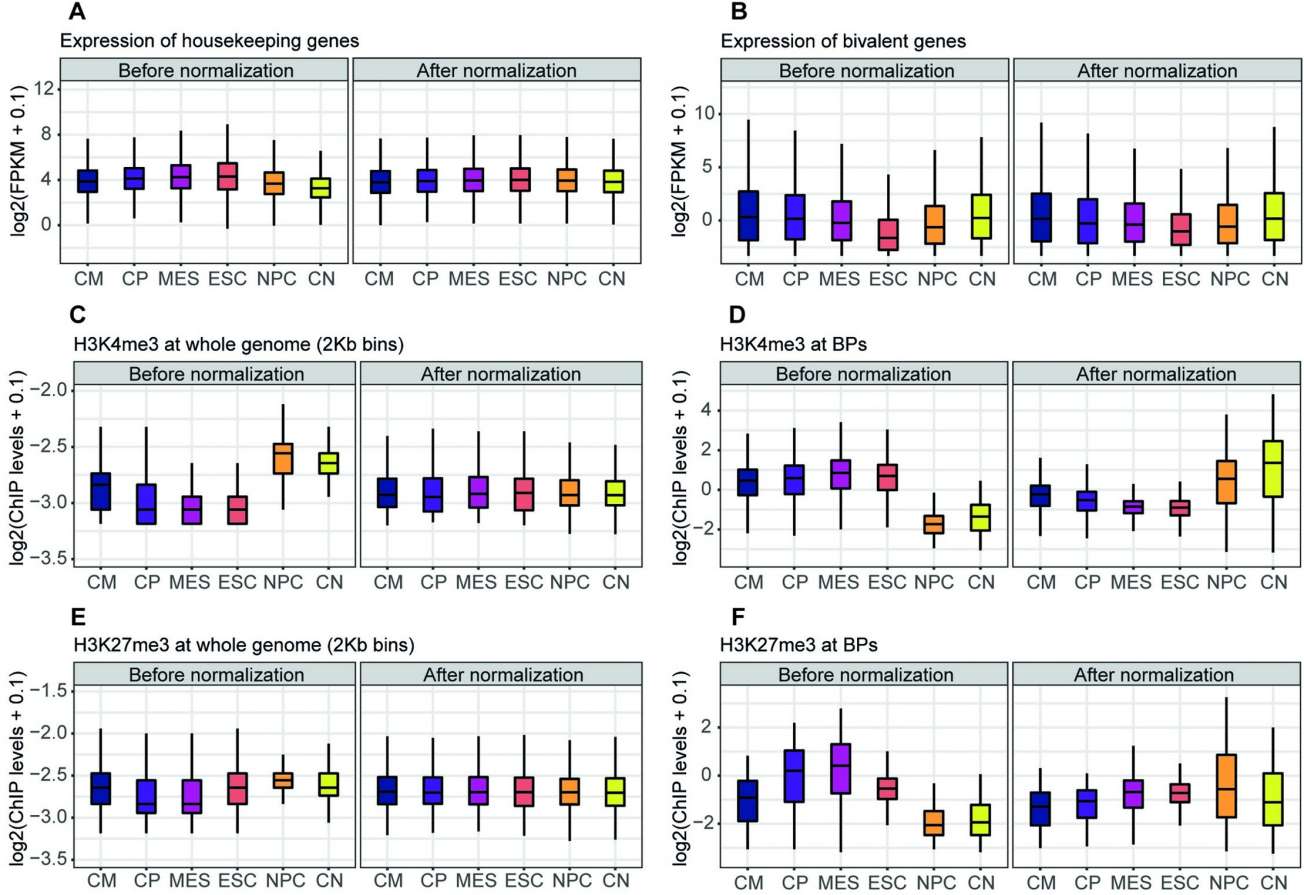
RNA-seq and ChIP-seq data before and after LOESS normalization. (A) Raw and normalized expression of 3,277 housekeeping genes along cardiac and neural differentiation from ESCs. (B) Raw and normalized expression of 3,459 bivalent genes along the same time points as A. (C) Raw and normalized H3K4me3 ChIP-seq signal levels at all 2-Kb bins of the genome. (D) Raw and normalized H3K4me3 ChIP-seq signal levels at 3,344 BPs. (E, F), same as C and D, respectively, but for H3K27me3. CM, cardiomyocytes; CN, cortical neurons; CP, cardio precursors; MES, mesoderm; NPC, neural precursors.

We first applied this normalization method over the expression of a set of 20,706 protein-coding genes in the mouse genome, at each differentiation time point, and ESCs (MA plots of each differentiation time point against ESCs before and after LOESS normalization are shown in S3A Fig). As LOESS normalization assumes that expression is equal in all samples, a general balance in global expression distribution of all time points is expected (S3B Fig). To further confirm the normalization efficiency, we tested its performance on two different subsets of genes: housekeeping genes and bivalent genes. We hypothesized that housekeeping genes would show a balanced distribution of expression after normalization, while bivalent genes would increase their expression globally during differentiation (as some of them are activated). For this, we extracted a list of mouse housekeeping genes across 14 mouse tissues from the literature [38] to check their expression. We also evaluated the normalization on our list of bivalent genes (e.g., those associated to BPs). Indeed, after LOESS normalization, the expression of housekeeping genes was correctly balanced (Fig 3A), whereas the expression of bivalent genes maintained the characteristic pattern of increased expression across time (Fig 3B).

We then ran the same normalization method on the ChIP-seq samples for H3K27me3, H3K4me3, H3K4me1, and H3K36me3 at the same time points (MA plots before and after LOESS normalization over the full set of bins of 2 Kb at Chr19 are shown in S4 Fig). Similar to expression data, we evaluated our normalization method for the H3K4me3 and H3K27me3 ChIP-seq levels across differentiation on two different sets of genomic regions: the whole collection of bins of 2 Kb in which the genome is segmented, and the coordinates of our collection of BPs. We hypothesized that global ChIP-seq levels of the whole genome would become balanced irrespectively of the particular histone modification analyzed, while BPs should present a different pattern for H3K4me3 and H3K27me3 (e.g., increase and decrease of signal along differentiation time points respectively, as a subset of the bivalent genes becomes activated during time). Indeed, after LOESS normalization, the levels of H3K4me3 along the whole genome were balanced (Fig 3C), whereas the same histone mark at BPs presented a clear pattern of increase during differentiation (Fig 3D). Notably, for H3K27me3, we observed the same balance in the levels along the whole genome after LOESS normalization (Fig 3E), while BPs exhibited a pattern of decreased signal across differentiation, in contrast to that observed for H3K4me3 (Fig 3F). In all cases, therefore, there is a substantial improvement after applying this normalization approach, while the analysis solely based on data before normalization would be misleading.

### Poised enhancers and bivalent promoters are good predictors of gene expression during differentiation

After normalizing the data on expression and histone modifications across differentiation, we next generated predictive models using PEs and BPs for each differentiation time point. We used the Hi-C–top interactions involving PEs, BPs, and target genes in ESCs (1,846 PEs and 1,382 BPs associated with 1,434 target genes). From this dataset, a total of 1,063 protein-coding genes were used in the analysis. As the number of genes is smaller than in the previous gene sets, we decided to build the models at each cell type from the full set of genes to be evaluated in the rest of the differentiation time points. This approach has the advantage of allowing us to check whether the relationship between gene expression and histone marks in PEs is universal. This would be true if a model trained in a specific cellular context has a good performance in predicting gene expression in another one. We hypothesized that, as shown previously for promoters and gene bodies [9–11,14], there is a universal relationship between gene expression and histone modifications at PEs.

**Fig 4:**
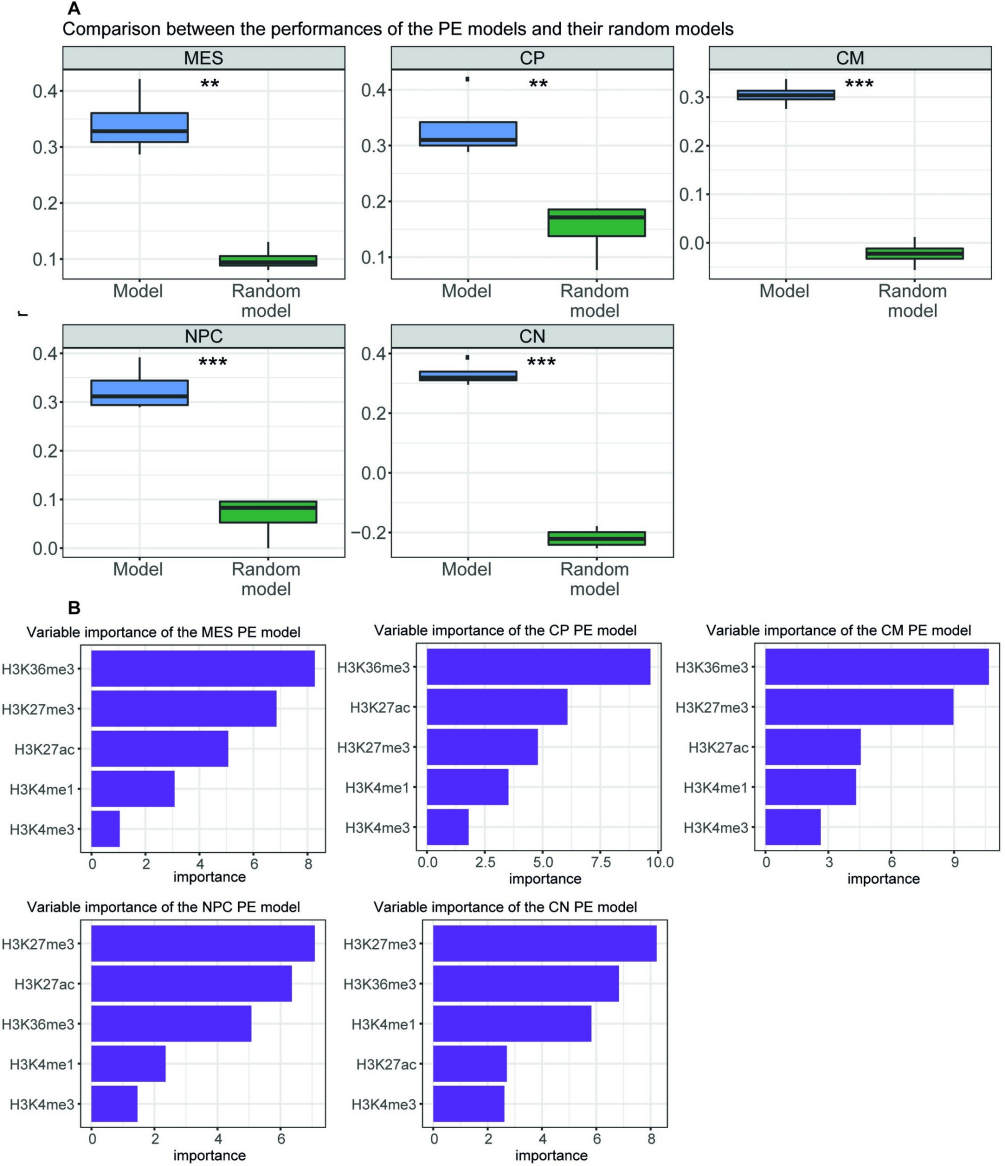
PE models trained in differentiation time points. (A) Performance of each differentiation enhancer model on the rest of the differentiation time points as compared to performance over random models. Performance is represented as Pearson’s correlation (*r*) between predicted expression and measured expression. Significance was assessed using a paired Student’s *t*-test between the performance of the models and the performance of the random models paired by the differentiation test set (****p < 0.0001, ***p < 0.001, **p < 0.01, *p < 0.05). CM, cardiomyocytes; CN, cortical neurons; CP, cardio precursors; MES, mesoderm; NPC, neural precursors. (B) Importance of the histone modifications for each differentiation enhancer model. Importance is defined as the contribution of each variable in the linear regression predictive model and corresponds to the absolute value of the t-statistic for each model parameter.

As a control, we randomized expression data and calculated predictive models for each time point. Next, we evaluated the performance of the randomized models on each differentiation dataset. We observed that predictive models for PEs and BPs obtained a significantly higher performance than randomized controls (Figs 4A, S5 and S6A). Surprisingly, all PE models achieved the best performance in cardiomyocytes (Table 1), suggesting that cardiomyocyte gene expression is easier to predict than the gene expression of the other time points. Moreover, all PE models had similar performances at each time point (Table 1). These observations are also true for the BP models (Table 2). Taken together, our results indicate that there is a universal quantitative relationship between gene expression and histone modifications at PEs and BPs across cardiac and neural differentiation.

**Table 1:** Performance of each PE differentiation model at every differentiation time point.

	MES	CP	CM	NPC	CN
MES model	-	0.34	0.42	0.32	0.29
CP model	0.3	-	0.42	0.32	0.29
CM model	0.3	0.34	-	0.31	0.28
NPC model	0.3	0.33	0.39	-	0.29
CN model	0.3	0.32	0.39	0.32	-

For each time point of cardiac (MES/CP/CM) and neural (NPC/CN) PE models (rows), the performance of the PE predictive models is shown for each cell type (columns). The performance values are represented as Pearson’s correlation (*r*) between the measured expression and the predicted one. CM, cardiomyocytes; CN, cortical neurons; CP, cardio precursors; MES, mesoderm; NPC, neural precursors.

**Table 2:** Performance of each BP differentiation model at every differentiation time point.

	MES	CP	CM	NPC	CN
MES model	-	0.78	0.77	0.66	0.77
CP model	0.74	-	0.79	0.67	0.72
CM model	0.72	0.78	-	0.65	0.71
NPC model	0.71	0.77	0.77	-	0.72
CN model	0.73	0.77	0.78	0.69	-

For each time point of cardiac (MES/CP/CM) and neural (NPC/CN) BP models (rows), the performance of the BP predictive models is shown for each cell type (columns). The performance values are represented as Pearson’s correlation (*r*) between the measured expression and the predicted one. CM, cardiomyocytes; CN, cortical neurons; CP, cardio precursors; MES, mesoderm; NPC, neural precursors.

In order to confirm the predictive capacity of distal PEs (>5 Kb from a TSS, a total of 696 PEs), we generated new distal PE models (S7 Fig). Indeed, distal PEs maintained the predictive capacity. In this case, 486 protein-coding genes were included in the modelling.

Finally, we assessed the variable importance of the PE models for identifying differences in the contribution of each histone modification to the predictive models in different cellular contexts (Fig 4B). Strikingly, we observed that, in general, the two most important variables are H3K27me3 and H3K36me3. H3K36me3 was the most important histone modification for cardiac differentiation, whereas H3K27me3 was the most important for neural differentiation. In general, H3K27ac followed the above-mentioned histone modifications. H3K4me1 had a relatively low relevance to the predictive models, which suggests that it is involved in delimitating the enhancer regions rather than in contributing to its function. H3K4me3, which was vastly associated with promoter activity, is accordingly the least informative mark for the prediction of gene expression using PEs, suggesting that H3K4me3 is not associated to enhancer activity. The variable importance in the BPs showed that H3K27me3, H3K27ac and, importantly, H3K4me3 were the most informative variables (S6B Fig). Our results suggest that the quantitative relationship between histone modifications varies according to their location in PEs or BPs. Critically, even though there is a universal quantitative relationship between histone modifications and gene expression, this relationship can vary depending on the cellular context.

As for ESCs, we also assessed the effect of H3K27me3 absence over H3K27ac importance in the current scenario. Therefore, we generated new PE predictive models without H3K27me3 (S8A Fig). Interestingly, H3K27ac was generally the most predictive variable for these predictive models, followed by H3K36me3 (S8B Fig). Therefore, we wondered whether in absence of H3K27ac, H3K27me3 would be also more important than H3K36me3. Indeed, when generating new PE models in absence of H3K27ac (S9A Fig), H3K27me3 had the highest importance in all the cases (S9B Fig). Therefore, H3K27me3 seems the most informative variable for predicting gene expression from PEs.

The importance of H3K36me3 seen in Fig 4B agrees with the fact that almost 60% of the PEs that entered the modelling are located within gene bodies. As H3K36me3 is located in the gene body of active genes [39,40], intragenic enhancers also become marked when the genes start to be expressed during differentiation. Therefore, we divided PEs into two groups, intragenic or intergenic, and built new PE predictive models. Both, intragenic and intergenic models were capable of predicting gene expression (S10A and S11A Figs). When assessing for variable importance, we observed that, as expected, H3K36me3 maintained its high contribution in the intragenic predictive models (S10B Fig). However, H3K36me3 importance was reduced in the intergenic predictive models (S11B Fig). On the contrary, H3K27me3 maintained its importance in both, intergenic and intragenic models. Thus, H3K27me3, and not H3K36me3, behaves as a truly universal predictor of PE activity.

### Poised enhancers and bivalent promoters are good predictors of gene expression in mouse embryonic tissues

**Fig 5:**
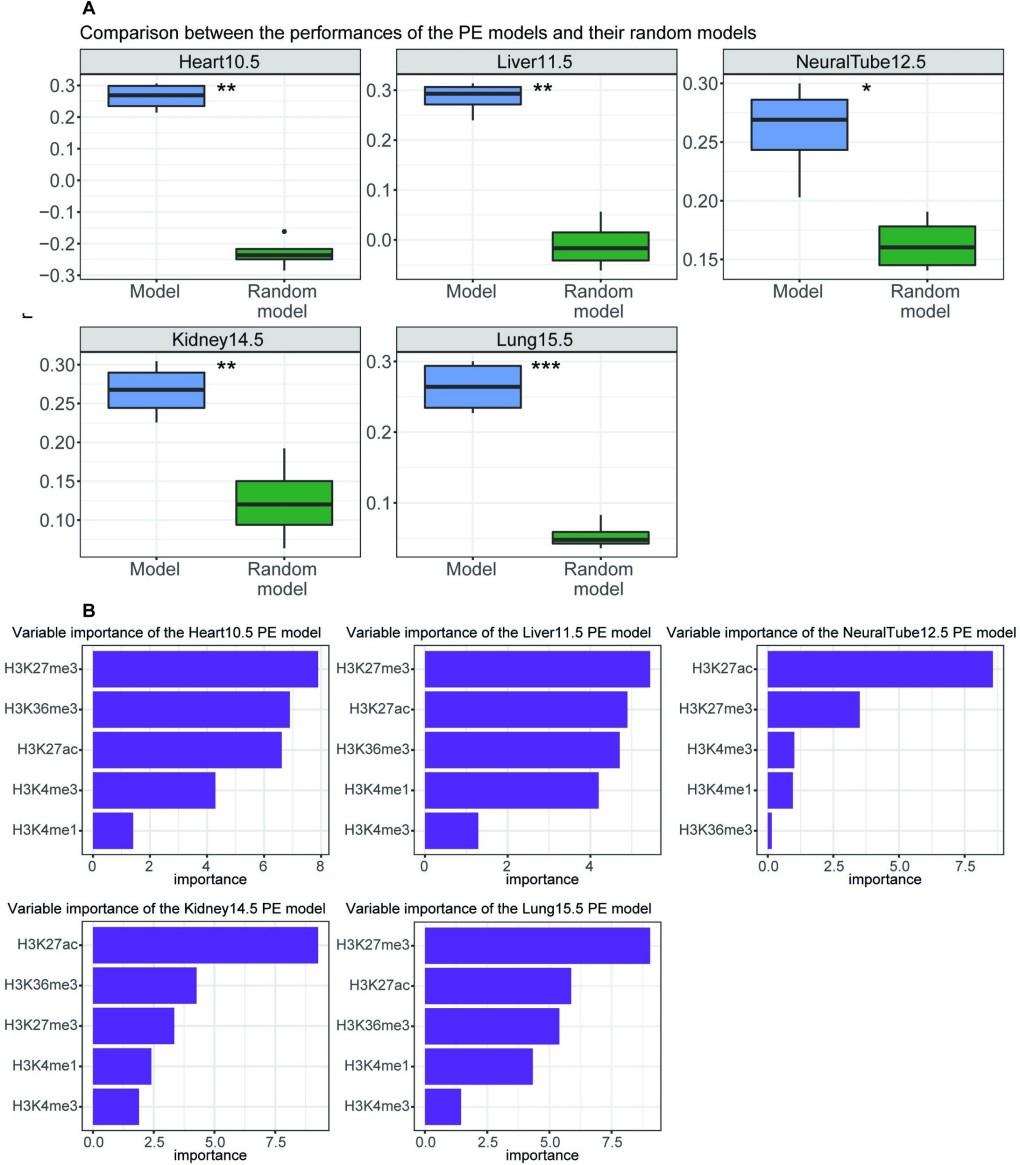
PE models trained using developmental stages. (A) Performance of each differentiation PE model on the rest of the developmental stages as compared to the performance over the random models. Performance is represented as Pearson’s correlation (*r*) between predicted expression and measured expression. Significance was assessed using a paired Student’s *t*-test of the performance of the models or of the random models paired by a differentiation test set (****p < 0.0001, ***p < 0.001, **p < 0.01, *p < 0.05). (B) Importance of histone modifications for each development PE model. Importance is defined as the contribution of each variable in the linear regression predictive model and corresponds to the absolute value of the t-statistics for each model parameter. Heart10.5, heart tissue from 10.5 embryonic day; Kidney14.5, kidney tissue from 14.5 embryonic day; Liver11.5, liver tissue from 11.5 embryonic day; Lung15.5, lung tissue from 15.5 embryonic day; NeuralTube12.5, neural tube tissue from 12.5 embryonic day.

In order to extend our findings from *in vitro* differentiation to *in vivo*, we learnt predictive PE and BP models from mouse developmental stages at different tissues. We downloaded ChIP-seq data of H3K27me3, H3K27ac, H3K36me3, H3K4me3 and H3K4me1 and RNA-seq on mouse embryos (heart tissue from 10.5 embryonic day, liver tissue from 11.5 embryonic day, neural tube tissue from 12.5 embryonic day, kidney tissue from 14.5 embryonic day, and lung tissue from 15.5 embryonic day) from ENCODE [41]. We first normalized the ChIP-seq and expression data following the LOESS approach. A total of 1,087 protein-coding genes entered the analysis. We observed that PEs were also predicting gene expression during mouse embryo development (Fig 5A). Again, when assessing for variable importance, we found that H3K27me3 was contributing the most, followed by H3K27ac and H3K36me3 (Fig 5B).

Next, we confirmed that BPs were also predicting gene expression during mouse embryo development (S12A Fig). In this case, the most predictive variable was H3K27ac followed by H3K27me3 and H3K4me3 (S12B Fig). Therefore, we further confirmed that differences in variable importance between PE models and BP models exist, which suggests that different histone modifications relate better to PE and BP function respectively.

## Discussion

To study the full spectrum of active and repressed enhancers, we used ESCs as a model system. Nevertheless, a third class of enhancers, termed intermediate or primed enhancers, exists in this cellular context; intermediate enhancers are decorated with H3K4me1 but lack both H3K27ac and H3K27me3 [21,42]. Originally, intermediate enhancers were classified as PEs [33]; however, more recent publications now use the term PE only for H3K27me3–marked enhancers [21,22,42–44]. The intermediate enhancer signature was found in our chromatin state model (state 5). We decided to focus only in AEs and PEs, though, as intermediate enhancers remain poorly understood, and their target promoters have not been unambiguously identified. In the near future, intermediate enhancers could be introduced in the modelling to explore their impact on the performance of the predictions and to discover new relationships between histone modifications at enhancers and gene expression. However, this is not a limitation for our differentiation predictive models. Here, enhancers and promoters are required to be in either a poised or a bivalent state in ESCs, but many will transition towards an active, or even intermediate state, along cardiac and neural *in vitro* differentiation, and along embryo development. Therefore, in the subset of PEs during differentiation, we have assessed the dynamics of a complete spectrum of enhancers in cardiac (mesoderm/cardio precursors/cardiomyocytes) and neural (neural precursors/cortical neurons) cells, and in developmental tissues (heart, liver, neural tube, kidney and lung). However, it is worth mentioning that our conclusions likely only apply to this system where PEs have been described. Therefore, further work on PEs in other cellular contexts will be necessary in order to generalize our findings.

One could speculate that the ESC enhancer model performance is an artefact of matching the chromatin state of promoters and enhancers (both active or both repressed). However, as no expression information is introduced in this step, further conclusions are not affected by this matching procedure. Moreover, we have confirmed the predictive capacity of PEs in the differentiation PE models, in which we do not require a coordinated activation of PEs and BPs. One could argue that the correlation coefficients obtained in the ESC enhancer models are a mere consequence of the bimodality observed in our scatterplots of gene expression prediction. However, in absence of H3K27me3 as a predictive variable, this bimodality disappears, and importantly, the correlation between predicted and measured expression is maintained (*r* = 0.36). Indeed, this correlation denotes the good performance of the model, and therefore, confirms the predictive power of histone modifications at enhancers. Thus, H3K27me3 could be the cause of the bimodality, probably because it is the histone modification that better differentiates between active and repressed enhancers in ESCs. Interestingly, no bimodality is observed in the scatterplots of the differentiation predictive models, which further confirms the predictive power of histone modifications at enhancers.

At the promoter level, the performance of ESCs and differentiation models was very similar (*r* = 0.79 vs. *r* ≈ 0.75). Our limitation of being only able to use a reduced number of histone modifications in the differentiation models (an issue that will be easily overcome when more ChIP-seq datasets are available) could be the reason for the minimal difference in the promoter models’ performance. Indeed, at the enhancer level, the difference in performance between ESCs and the differentiation models was higher (*r* = 0.49 vs. *r* > 0.3). Other factors besides the number of ChIP-seq datasets used could explain such a difference: (i) as mentioned before, enhancers and promoters in the ESC model were required to match their chromatin state, which could lead to overrating the performance of the ESC enhancer model; (ii) the enhancer-promoter Hi-C interactions were taken from data published on ESCs. Nevertheless, we predicted gene expression in cellular contexts distinct from ESCs; (iii) some genes might be specific for a cell lineage, and their interactions with enhancers might be lost in the other cell lineages. In these cases, the enhancer and the gene would no longer be related; (iv) enhancer–promoter interactions relevant for early stages of differentiation might be lost once they have served their function. This would imply that the enhancer and its target gene are no longer coordinated. In fact, it has been shown that intensive rearrangement of promoter–enhancer interactions occurs during differentiation, and that these loops become disrupted when their target genes are repressed [30,31].

Moreover, one could argue that the linear regression approach used in this study might be a too generic model, which could be the reason for lower performance of the enhancer models when compared to promoter models. Thus, we re-analyzed our collection of enhancer-gene associations in the Hi-C-top dataset with other methodologies, besides linear regression, which are conceptually more complex. In all cases, although more time-consuming, the performance of these methods did not improve our initial result (Table 3). We believe that the difference in performance between promoter (including BP) and enhancer (including PE) models could be due to: (i) the difficulties of assigning enhancers to their target genes genome-wide, which can lead to incorrect associations; and (ii) the more complex gene expression regulation by enhancers, when more than one enhancer–with different levels (or type) of histone marks–can regulate the same gene.

**Table 3:** Comparative analysis of other methodologies to predict gene expression in the Hi-C-top dataset.

Model	method value in caret R package [45]	Performance (r)	Most important variable
Neural Network	neuralnet	0.48	H3K27me3
Lasso	lasso	0.48	H3K27me3
Random Forest	rf	0.44	H3K27me3
Support Vector Machines with Linear Kernel	svmLinear	0.48	H3K27me3
Principal Component Analysis	pcr	0.46	H3K27me3
Linear regression (used in this work)	lm	0.49	H3K27me3

Predictive models were obtained using default parameters. Variable importance was assessed with varImp function from caret R package [45].

How to assign enhancers to target genes is still under debate. In this study, we used Hi-C and matched chromatin states to link enhancers to genes and promoters. A recent study evaluated distinct ways of linking genes to enhancers by modelling gene expression and DNase-seq data [17]. They showed that expression predictive models using chromatin conformation data, such as Hi-C, performed better than those using other traditional ways of assigning target genes, such as the closest-gene method or by distance. The closest-gene method consists of assigning each enhancer to the nearest TSS. This prevents enhancers from being assigned to two or more genes but does not take into account that one enhancer can regulate the expression of more than one gene [46,47]. The distance method consists of assigning an enhancer to all the genes that are closer than a pre-set number of base pairs. We achieved a performance of *r* = 0.34 by using 1 Mb distance to assign enhancers to promoters. Although this predictive model had lower performance than the models based on Hi-C data (*r* = 0.38 and *r* = 0.49 for Hi-C–all and Hi-C–top enhancer models, respectively), it maintained the predictive capacity. This suggests that in absence of Hi-C data, using 1 Mb distance to assign target genes to enhancers performs well. Moreover, data from other chromatin capture techniques could be useful to associate enhancers to promoters as well. Indeed, preliminary results using promoter capture Hi-C to associate enhancers to promoters improved the performance of our enhancer predictive models in comparison to those in which Hi-C data was used. We argue that this gain in the performance of gene expression prediction is likely due to promoter capture Hi-C enriching for the best interactions.

Apart from 3C techniques, two novel computational methods have been developed to properly identify enhancer–gene associations using chromatin capture data (such as Hi-C and Hi-ChIP) and enhancer activity data (such as H3K27ac ChIP-seq and DHS-seq) [19,48]. For instance, the so-called FOCS inference method provides a map of active enhancer–promoter associations consistent across several cellular contexts, although no cell-type specific associations could be detected [48]. Further, the activity-by-contact method identified cell-type specific associations of active enhancers and genes [19]. However, neither methodology can be applied to PEs due to the lack of enhancer activity. Indeed, the capacity of PEs to dynamically predict variable gene expression during differentiation suggests that our approach of assigning target genes to PEs performs properly in this context.

We have reported differences in the histone modification contribution to the expression predictive models depending on their location in enhancers or promoters. The different contribution of each histone modification suggests that the epigenetic landscape is different in enhancers and promoters. For example, although H3K4me3 has been previously shown to be located in enhancers [25–28], our results suggest that its presence in enhancers has little association to gene expression. Therefore, H3K4me3 does not seem to be a good indicator of enhancer activity. In contrast, H3K4me3 proved to be key in predicting gene expression from the differentiation BP models, confirming its relevance in establishing promoter activity. Moreover, whereas H3K36me3 proved to be important for the differentiation PE models–mainly those intragenic–, it showed little contribution to the BP ones. Even though there is a universal relationship between histone modifications and gene expression, we observed that H3K36me3 is more informative in the cardiac PE models than in the neural PE models. We reached this conclusion thanks to our LOESS normalization approach, which allowed us to reduce biases in all datasets used (RNA-seq and ChIP-seq), such that our results were not influenced by the different origin of data. Without such a normalization, the conclusions reached would be wrong. However, it is worth mentioning that we assume constancy of ChIP-seq signal and expression, although they might change in their abundance during differentiation. This problem will be solved in the future with spike-in normalization. Strikingly, H3K27me3 was found to be the most important histone modification in the majority of predictive models for enhancers and promoters. This suggests that H3K27me3 plays a key role in gene regulation, as it is important for both types of regulatory regions. Our results show that mainly H3K27me3, and also in combination with H3K36me3 and H3K27ac, are sufficient to predict future gene expression from PEs. In any case, the predictive power of our models will benefit in the future from the introduction of other histone modifications into the modelling, which can be extremely useful for identifying unknown quantitative relationships between histone modifications at enhancers and gene expression.

Finally, other types of information could also be introduced in the modelling in the future. In fact, previous work has modelled gene expression using accessibility data (e.g. DHS-seq) [14,16,17], and other types of ChIP-seq samples (e.g. TFs or RNA polymerase II) [10,11,13,49]. It would be also interesting to use enhancer RNA (eRNA) data to predict gene expression of target genes. Promising results have been obtained in predicting eRNA transcription by modelling GRO-seq and histone modification ChIP-seq at enhancers [50]. Indeed, Pearson’s correlation between PRO-seq [51] signal and H3K27ac ChIP-seq signal at intergenic enhancers in ESC is 0.41, which further supports that eRNA expression might be a good predictor, probably similar to H3K27ac. All this information at enhancers could be integrated into the modelling to improve the power and, most importantly, to discover new quantitative relationships between gene expression and multiple epigenetic features.

## Materials and methods

### Cell culture

E14Tg2A ESCs were cultured feeder-free on 15-cm plates coated with 0.1% gelatin. Plates were coated with gelatin for 15 min at 37°C, and then non-bound gelatin was removed. ESCs were cultured with Glasgow minimum essential medium (Sigma) supplemented with β-mercaptoethanol, sodium pyruvate, penicillin–streptomycin, non-essential amino acids, GlutaMAX, 20% fetal bovine serum (Hyclone), and leukemia inhibitory factor (LIF).

### Chromatin immunoprecipitation

Cells were grown in 15-cm plates until 70% confluency and crosslinked in 1% formaldehyde in growth medium for 10 min at room temperature in a shaker. To stop fixation, glycine was added to a final concentration of 0.125 M and incubated for 5 min at room temperature. Cells were then washed twice with ice-cold PBS and harvested by gently scrapping plates (on ice) in PBS plus protease inhibitors. Cells from two 15-cm plates were pooled together and centrifuged at 3,400 × *g* at 4°C for 5 min. Cell pellets were frozen at –80°C until use.

Chromatin was prepared by resuspending the crosslinked pellet in 1.3 ml ice cold ChIP buffer [1× volume SDS buffer (100 mM NaCl, 50 mM Tris-HCl pH 8.1, 5 mM EDTA pH 8.0, and 0.5% SDS) and 0.5 × volume Triton dilution buffer (100 mM NaCl, 100 mM Tris-HCl pH 8.6, 5 mM EDTA pH 8.0, and 5% Triton X-100)] plus proteinase inhibitors. Samples were sonicated 40 cycles (30 seconds on/30 seconds off) in a Bioruptor Pico (Diagenode) and centrifuged at 16,000 × *g* at 4°C for 20 min to remove the cell debris. To check chromatin size, a 25-μl aliquot was mixed with 175 μl of PBS plus 5 μl of 20 mg/ml proteinase K, and de-crosslinked for 5 h at 65°C. DNA was purified using the QIAquick PCR purification kit (Qiagen), quantified in Nanodrop, and checked by electrophoresis on a 1.2% agarose gel.

ChIP experiments were performed using 30 μg of chromatin (DNA) and 5 μg of antibody in a final volume of 500 μl ChIP buffer. Aliquots of 5 μl were removed as input material (1%). ChIP samples were incubated overnight at 4°C on rotation, and then Protein A agarose beads (Diagenode) (42 μl per ChIP) were blocked 30 min with 0.05% BSA, washed, and added to the ChIP reaction. Samples were incubated for 2 h at 4°C with rotation. After incubation, beads were washed three times with 1 ml of low-salt buffer (140 mM NaCl, 50 mM HEPES pH 7.5, and 1% Triton X-100) and once with 1 ml high-salt buffer (500 mM NaCl, 50 mM HEPES pH 7.5, and 1% Triton X-100). ChIPed material was eluted from the beads in 200 μl freshly prepared elution buffer (1% SDS, 100 mM NaHCO3) at 65°C in a shaker (1000 rpm) for 1 h. Input samples were also brought to 200 μl with elution buffer. After addition of 8 μl of 5 M NaCl to the eluted chromatin and input samples, samples were de-crosslinked overnight at 65°C. The next day, samples were treated with proteinase K [1 μl of 20 mg/ml Proteinase K, plus 4 μl 0.5 M EDTA, and 8 μl Tris-HCl pH 6.5] for 1 h at 45°C. ChIPed DNA and inputs were purified using the QIAquick PCR purification kit (Qiagen) and eluted in 60 μl. The following antibodies were used in the ChIP experiments: H3K27me3 (Millipore, #07–449); H3K4me3 (Diagenode, C15410003); H3K4me1 (Abcam, ab8895); H3K27Ac (Millipore, #07–360); H3 (Abcam, Ab1791); H3K36me3 (Abcam, ab9050); H3K27me1 (Active Motif, #61015); H3K27me2 (Cell Signaling, #9728); H3K79me2 (Abcam, ab3594); H2Bub (Cell Signaling, #5546); and H4K20me3 (Abcam, ab9053). Library preparation for ChIP-seq experiments was performed at the UPF/CRG Genomics Unit. Libraries were sequenced using Illumina HiSeq2000 sequencer.

### Input datasets

Raw files and processed data from experiments performed in this study are available at the Gene Expression Omnibus (GEO) under the accession number GSE150633. Raw data of multiple samples from the literature was downloaded and reanalyzed to be included in the study. RNA-seq data of mouse ESCs was extracted from a previous publication from our lab (GEO accession number: GSE79606) [23]. ChIP-seq data of p300 in ESCs was obtained via GEO (GEO accession number: GSE89211) [21]. ChIP-seq data of H3K27me3, H3K4me3, H3K27ac, H3K4me1, and H3K36me3, and RNA-seq data of cardiac differentiation (mesoderm, cardioprecursors and cardiomyocytes), were obtained from https://b2b.hci.utah.edu/gnomex/ (accession numbers: 44R and 7R2) [35]. ChIP-seq data of H3K27me3, H3K4me3, H3K27ac, H3K4me1, and H3K36me3, RNA-seq data of neural differentiation (neural precursors and cortical neurons), and Hi-C data of ESCs were retrieved from GEO (GEO accession number: GSE96107) [30]. PRO-seq data of mouse ESC was obtained from a previous publication from our lab (GEO accession number: GSE99530) [51]. ChIP-seq data of H3K27me3, H3K4me3, H3K27ac, H3K4me1, and H3K36me3, and RNA-seq of mouse developmental stages (heart tissue from 10.5 embryonic day, liver tissue from 11.5 embryonic day, neural tube tissue from 12.5 embryonic day, kidney tissue from 14.5 embryonic day, lung tissue from 15.5 embryonic day) were obtained from ENCODE project [41]. The list of ENCODE accession numbers can be found in S10 Table. When replicates were available, pooling was done except for the ChIP-seq samples of H3K4me3 of neural precursors (replicate 1 was used) and H3K27ac of neural precursors (replicate 2 was used).

### ChIP-seq analysis

The sequence reads of ChIP-seq data were mapped to the mm10 version of the mouse genome with the BOWTIE software [52], setting the option–m 1, which eliminates reads that align in more than one region. The ChIP-seq profiles were obtained using the function buildChIPprofile from SeqCode (https://github.com/eblancoga/seqcode). For the p300 ChIP-seq, peak calling against input was performed using MACS [53] with the option—shiftsize 100, which shifts tags to their midpoint. Information about the total number of reads and read mapping of each ChIP-seq experiment produced in this study can be found in Table 4.

**Table 4:** Information on ChIP-seq experiments produced in this study.

ChIP-seq	Total reads	Mapped reads	Multilocus reads
**H2Bub**	42268079	32671442 (77.30%)	7633345 (18.06%)
**H3K4me1**	43515038	35408178 (81.37%)	6365630 (14.63%)
**H3K4me3**	45129019	34342716 (76.10%)	9253789 (20.51%)
**H3K27me1**	45349251	26830739 (59.16%)	15003526 (33.08%)
**H3K27me2**	60609374	44651269 (73.67%)	12963849 (21.39%)
**H3K27me3**	37386877	26823175 (71.74%)	8234058 (22.02%)
**H3K27ac**	35210421	25105518 (71.30%)	8368750 (23.77%)
**H3K36me3**	42562036	28957647 (68.04%)	11123530 (26.13%)
**H3K79me2**	60011275	43270733 (72.10%)	13458082 (22.43%)
**H4K20me3**	34180446	16175574 (47.32%)	15452266 (45.21%)
**H3**	53276040	35997064 (67.57%)	14569719 (27.35%)
**Input**	41583841	28857996 (69.40%)	10598866 (25.49%)

### Chromatin segmentation

ChromHMM [54] was used to obtain a chromatin segmentation model for ESCs using the default parameters. The input data were ChIP-seq experiments of H3K4me3, H3K27me3, H3K27ac, and H3K4me1, using ChIP-seq of H3 as control. First, the function BinarizeBam was used to binarize the input mapped data. Next, the LearnModel function was ran to learn different chromatin segmentation models of ESCs, using from 4 to 16 states; the 9-state model was selected because it showed the higher number of states with no redundancy.

### RNA-seq analysis

The pair-end sequence reads of RNA-seq data were mapped to the mm10 version of the mouse genome with TopHat [55], setting the options—mate-inner-dist 100, which is the expected mean distance between mate pairs, and -g 1, which eliminates those reads which align in more than one region. The RNA-seq profiles were obtained using the function buildChIPprofile from SeqCode. The FPKMs (fragments per kilobase of transcript per million mapped reads) of each gene in the RefSeq catalogue [24] of the mouse genome were calculated using Cufflinks [56], setting the option—max-bundle-frags 5,000,000, which specifies the maximum genomic length for the bundles.

### PRO-seq analysis

The single-end sequence reads of PRO-seq data was mapped to the mm10 version of the mouse genome with TopHat [55], setting the options—mate-inner-dist 100, which is the expected mean distance between mate pairs, and -g 1, which eliminates those reads which align in more than one region. The normalized count of reads of PRO-seq at intergenic enhancers averaged by the length of the region was calculated by recoverChIPlevels from SeqCode.

### Hi-C analysis

Hi-C data were processed with TADbit [57]. Briefly, sequencing reads were mapped to the reference genome (mm10) by applying a fragment-based strategy, which is dependent on the GEM mapper [58]. Mapped reads were filtered to remove those resulting from unspecified ligations, errors, or experimental artefacts. Specifically, seven different filters were applied using the default parameters in TADbit: self-circles, dangling ends, errors, extra dangling-ends, over-represented, duplicated, and random breaks [57]. After pooling replicates, Hi-C data were normalized with OneD correction [59] at 5 kb of resolution to remove known biases. Significant Hi-C interactions were called with the analyzeHiC function of HOMER software suit [60], binned at 5 kb of resolution, and with the default *p*-value threshold of 0.001.

### Gene expression predictive model

The regression linear models were built to predict gene expression by adjusting the following formula:

$$y_{i}∼\beta_{0}+\beta_{1}\;x_{i1}+\cdots +\beta_{n}\;x_{in}+\epsilon$$

where *y*_*i*_ is the log_2_ of the FPKMs of gene *i*, with a pseudo count of 0.1. *x*_*i1*_ to *x*_*in*_ are the log_2_-normalized count of reads of each ChIP-seq signal at the defined promoters or enhancers averaged by the length of the region calculated by recoverChIPlevels from SeqCode, plus a pseudo count of 0.1. *β*_*0*_ to *β*_*n*_ are the coefficients that we would like to calculate and ε is the error. The predictive models were trained on protein-coding genes. The set of data was randomly divided into two subsets, a training subset with the 80% of entries, and a test subset with the remaining 20% of entries. In the case of differentiation, each of the time points was used as training subsets and then the predictive models were evaluated in the rest. A 10-fold cross-validation was repeat three times to verify that the quantitative relationship between expression and histone modifications was not specific for a subset of the data. The following functions were used: trainControl to perform the 10-fold cross-validation, train to train the models, and varImp to calculate the variable importance, from the R package caret [45]. For models trained on enhancers, genes were introduced into the dataset as many times as the number of associated enhancers they had. To evaluate the specificity of our predictive models, we randomly shuffled the expression values of all the genes in the mouse genome. Thus, for each initial predictive model a random model was also obtained, where all the values *y*_*i*_ were shuffled, maintaining the values of *x*_*i1*_ to *x*_*in*_ intact. This operation generates a new table of gene expression assignments in which the putative relationship between histone marking and expression of genes (if any) is completely lost. The random models were next generated following the same procedure as the models.

### LOESS normalization

The FPKMs of all protein-coding genes and ChIP-seq levels of PEs and BPs were normalized for ESCs, cardiomyocytes, cortical neurons, cardio precursors, mesoderm, and neural precursors; and also, for heart tissue from 10.5 embryonic day, liver tissue from 11.5 embryonic day, neural tube tissue from 12.5 embryonic day, kidney tissue from 14.5 embryonic day, and lung tissue from 15.5 embryonic day. To normalize the ChIP-seq levels of H3K27me3, H3K4me3, H3K27ac, H3K4me1, and H3K36me3 on the PEs and BPs, the genome was first divided into 2 Kb bins (note that bin size reflects the average size of PEs and BPs). Next, the count of reads, normalized by total number of reads and averaged by the length, was calculated with recoverChIPlevels function from SeqCode. Finally, the normalization parameters were calculated in those bins and applied to the count of reads normalized by the total number of reads and averaged by the length of PEs and BPs. The normalize.loess function of the R package affy [61] was used to normalize ChIP-seq data and expression data. Genes and bins with a 0 in any columns were discarded, as it was not possible to determine whether it was due to a sequencing error or a real absence of signal.

## Supporting information

S1 Fig Functional regions are covered by more than one class of state. (A) Segments of active states 1–4 cover the same functional regions delimited by peaks of H3K4me3, H3K27ac and H3K4me1. Differences in the definition of active states are due to the shape of the peaks over the same functional elements. The screenshot was taken from the UCSC Genome Browser [62]. (B) Segments of repressed states 6 and 7 denote the sharp peaks of H3K4me3 and H3K4me1 found inside broad regions covered by H3K27me3. State 8 corresponds to the fraction of H3K27me3 peaks that does not overlap with the other two marks. Differences in the definition of repressed states 6 and 7 are due to the shape of the peaks over the same functional elements. The screenshot was taken from the UCSC Genome Browser [62].
(TIF)

S2 Fig Performance of enhancer and promoter predictive models in ESCs. Predicted expression of the test subset of genes calculated by the models versus their measured expression by RNA-seq. Model performances are represented by the Pearson’s correlation (*r*) between predicted and measured expression values. (A) Left, the model trained on the promoter regions associated to at least one enhancer using all significant interactions of Hi-C (Hi-C–all promoter model). Right, the performance of the same model after randomizing the expression of the training subset of genes. The color bar represents the density of dots. (B) Left, the model trained on the enhancer regions associated to at least one promoter using all the significant interactions of Hi-C (Hi-C–all enhancer model). Right, the performance of the same model after randomizing the expression of the training subset of genes. The color bar represents the density of dots. (C) As for B, but using 1 Mb distance to connect enhancers to promoters. (F) As for B, but using from the Hi-C–top interactions, only distal enhancers (> 5 Kb from a TSS) to generate the model.
(TIF)

S3 Fig Performance of LOESS normalization in RNA-seq data. (A) MA plot before and after normalization of expression data at each differentiation time point against ESCs. M represents the log_2_ ratio of the intensities of the two samples and A is the log_2_ of the average intensity. Intensity is determined in FPKMs. After normalization, the regression line tends to M = 0. The color bar represents the density of dots. (B) Boxplot of expression of 15,065 protein-coding genes before and after LOESS normalization. CM, cardiomyocytes; CN, cortical neurons; CP, cardio precursors; MES, mesoderm; NPC, neural precursors.
(TIF)

S4 Fig Performance of LOESS normalization in the ChIP-seq data. MA plots before and after normalization of each differentiation time point against ESCs. M represents the log_2_ ratio of the intensities of the two samples, and A is the log_2_ of the average intensity. Intensity corresponds to normalized count of reads by total number of reads of the ChIP-seq samples of (A) H3K27me3, (B) H3K4me3, (C) H3K27ac, (D) H3K4me1, and (E) H3K36me3. The color bars represent the density of dots. CM, cardiomyocytes; CN, cortical neurons; CP, cardio precursors; MES, mesoderm; NPC, neural precursors.
(TIF)

S5 Fig Performance of PE differentiation models. Predicted expression of the test differentiation time points calculated by the models versus their measured expression by RNA-seq. Model performances are represented by the Pearson’s correlation (*r*) between predicted and measured expression values. The color bars represent the density of dots. (A) Model trained in mesoderm. (B) Model trained in cardio precursors. (C) Model trained in cardiomyocytes. (D) Model trained in neural precursors. (E) Model trained in cortical neurons. CM, cardiomyocytes; CN, cortical neurons; CP, cardio precursors; MES, mesoderm; NPC, neural precursors.
(TIF)

S6 Fig BP models trained using differentiation time points. (A) Performance of each differentiation BP model on the rest of the differentiation time points as compared to the performance over the random models. Performance is represented as Pearson’s correlation (*r*) between predicted expression and measured expression. Significance was assessed using a paired Student’s *t*-test of the performance of the models or of the random models paired by a differentiation test set (****p < 0.0001, ***p < 0.001, **p < 0.01, *p < 0.05). (B) Importance of histone modifications for each differentiation BP model. Importance is defined as the contribution of each variable in the linear regression predictive model and corresponds to the absolute value of the t-statistics for each model parameter. CM, cardiomyocytes; CN, cortical neurons; CP, cardio precursors; MES, mesoderm; NPC, neural precursors.
(TIF)

S7 Fig Distal PE models trained using differentiation time points. Performance of each differentiation BP model on the rest of the differentiation time points as compared to the performance over the random models. Performance is represented as Pearson’s correlation (r) between predicted expression and measured expression. Significance was assessed using a paired Student’s t-test of the performance of the models or of the random models paired by a differentiation test set (****p < 0.0001, ***p < 0.001, **p < 0.01, *p < 0.05). CM, cardiomyocytes; CN, cortical neurons; CP, cardio precursors; MES, mesoderm; NPC, neural precursors.
(TIF)

S8 Fig PE models trained using differentiation time points without H3K27me3 as predictive variable. (A) Performance of each differentiation model without H3K27me3 as predictive variable on the rest of the differentiation time points as compared to the performance over the random models. Performance is represented as Pearson’s correlation (*r*) between predicted expression and measured expression. Significance was assessed using a paired Student’s *t*-test of the performance of the models or of the random models paired by a differentiation test set (****p < 0.0001, ***p < 0.001, **p < 0.01, *p < 0.05). (B) Importance of histone modifications for each differentiation intragenic model. Importance is defined as the contribution of each variable in the linear regression predictive model and corresponds to the absolute value of the t-statistics for each model parameter. CM, cardiomyocytes; CN, cortical neurons; CP, cardio precursors; MES, mesoderm; NPC, neural precursors.
(TIF)

S9 Fig PE models trained using differentiation time points without H3K27ac as predictive variable. (A) Performance of each differentiation model without H3K27ac as predictive variable on the rest of the differentiation time points as compared to the performance over the random models. Performance is represented as Pearson’s correlation (*r*) between predicted expression and measured expression. Significance was assessed using a paired Student’s *t*-test of the performance of the models or of the random models paired by a differentiation test set (****p < 0.0001, ***p < 0.001, **p < 0.01, *p < 0.05). (B) Importance of histone modifications for each differentiation intragenic model. Importance is defined as the contribution of each variable in the linear regression predictive model and corresponds to the absolute value of the t-statistics for each model parameter. CM, cardiomyocytes; CN, cortical neurons; CP, cardio precursors; MES, mesoderm; NPC, neural precursors.
(TIF)

S10 Fig Intragenic PE models trained using differentiation time points. (A) Performance of each differentiation intragenic model on the rest of the differentiation time points as compared to the performance over the random models. Performance is represented as Pearson’s correlation (*r*) between predicted expression and measured expression. Significance was assessed using a paired Student’s *t*-test of the performance of the models or of the random models paired by a differentiation test set (****p < 0.0001, ***p < 0.001, **p < 0.01, *p < 0.05). (B) Importance of histone modifications for each differentiation intragenic model. Importance is defined as the contribution of each variable in the linear regression predictive model and corresponds to the absolute value of the t-statistics for each model parameter. CM, cardiomyocytes; CN, cortical neurons; CP, cardio precursors; MES, mesoderm; NPC, neural precursors.
(TIF)

S11 Fig Intergenic PE models trained using differentiation time points. (A) Performance of each differentiation intergenic model on the rest of the differentiation time points as compared to the performance over the random models. Performance is represented as Pearson’s correlation (*r*) between predicted expression and measured expression. Significance was assessed using a paired Student’s *t*-test of the performance of the models or of the random models paired by a differentiation test set (****p < 0.0001, ***p < 0.001, **p < 0.01, *p < 0.05). (B) Importance of histone modifications for each differentiation intergenic model. Importance is defined as the contribution of each variable in the linear regression predictive model and corresponds to the absolute value of the t-statistics for each model parameter. CM, cardiomyocytes; CN, cortical neurons; CP, cardio precursors; MES, mesoderm; NPC, neural precursors.
(TIF)

S12 Fig BP models trained using developmental stages. (A) Performance of each differentiation BP model on the rest of the developmental stages as compared to the performance over the random models. Performance is represented as Pearson’s correlation (*r*) between predicted expression and measured expression. Significance was assessed using a paired Student’s *t*-test of the performance of the models or of the random models paired by a differentiation test set (****p < 0.0001, ***p < 0.001, **p < 0.01, *p < 0.05). (B) Importance of histone modifications for each development BP model. Importance is defined as the contribution of each variable in the linear regression predictive model and corresponds to the absolute value of the t-statistics for each model parameter. Heart10.5, heart tissue from 10.5 embryonic day; Kidney14.5, kidney tissue from 14.5 embryonic day; Liver11.5, liver tissue from 11.5 embryonic day; Lung15.5, lung tissue from 15.5 embryonic day; NeuralTube12.5, neural tube tissue from 12.5 embryonic day.
(TIF)

S1 Table List of active promoters and target genes. Coordinates of the identified active promoters and their target genes (genome assembly mm10).
(XLSX)

S2 Table List of bivalent promoters and target genes. Coordinates of the identified bivalent promoters and their target genes (genome assembly mm10).
(XLSX)

S3 Table List of active enhancers. Coordinates of the identified active enhancers (genome assembly mm10).
(XLSX)

S4 Table List of poised enhancers. Coordinates of the identified poised enhancers (genome assembly mm10).
(XLSX)

S5 Table List of active enhancers, associated active promoters, and target genes (Hi-C–all). Coordinates of the identified active enhancers (*_e), associated active promoters (*_p), and target genes (genome assembly mm10). The association was done using all significant Hi-C interactions (Hi-C–all).
(XLSX)

S6 Table List of poised enhancers, associated bivalent promoters, and target genes (Hi-C–all). Coordinates of the identified poised enhancers (*_e), associated bivalent promoters (*_p), and target genes (genome assembly mm10). The association was done using all significant Hi-C interactions (Hi-C–all).
(XLSX)

S7 Table Model predictors. Coefficient and *p*-value of every predictor in each predictive model generated in this study.
(XLSX)

S8 Table List of active enhancers, associated active promoters, and target genes (Hi-C–top). Coordinates of the identified active enhancers (*_e), associated active promoters (*_p), and target genes (genome assembly mm10). The association was done using the top significant Hi-C interactions (Hi-C–top).
(XLSX)

S9 Table List of poised enhancers, associated bivalent promoters, and target genes (Hi-C–top). Coordinates of the identified poised enhancers (*_e), associated bivalent promoters (*_p) and target genes (genome assembly mm10). The association was done using the top significant Hi-C interactions (Hi-C–top).
(XLSX)

S10 Table List of ENCODE accession numbers. Accession numbers of the mouse embryo development data used in this study. Heart10.5, heart tissue from 10.5 embryonic day; Kidney14.5, kidney tissue from 14.5 embryonic day; Liver11.5, liver tissue from 11.5 embryonic day; Lung15.5, lung tissue from 15.5 embryonic day; NeuralTube12.5, neural tube tissue from 12.5 embryonic day.
(XLSX)
