# Supplementary figures and images for: Differential contribution to gene expression prediction of histone modifications at enhancers or promoters
Source: PLoS Comput Biol. 2021 Sep 2;17(9):e1009368. doi: 10.1371/journal.pcbi.1009368 (PMC8443064; doi:10.1371/journal.pcbi.1009368)

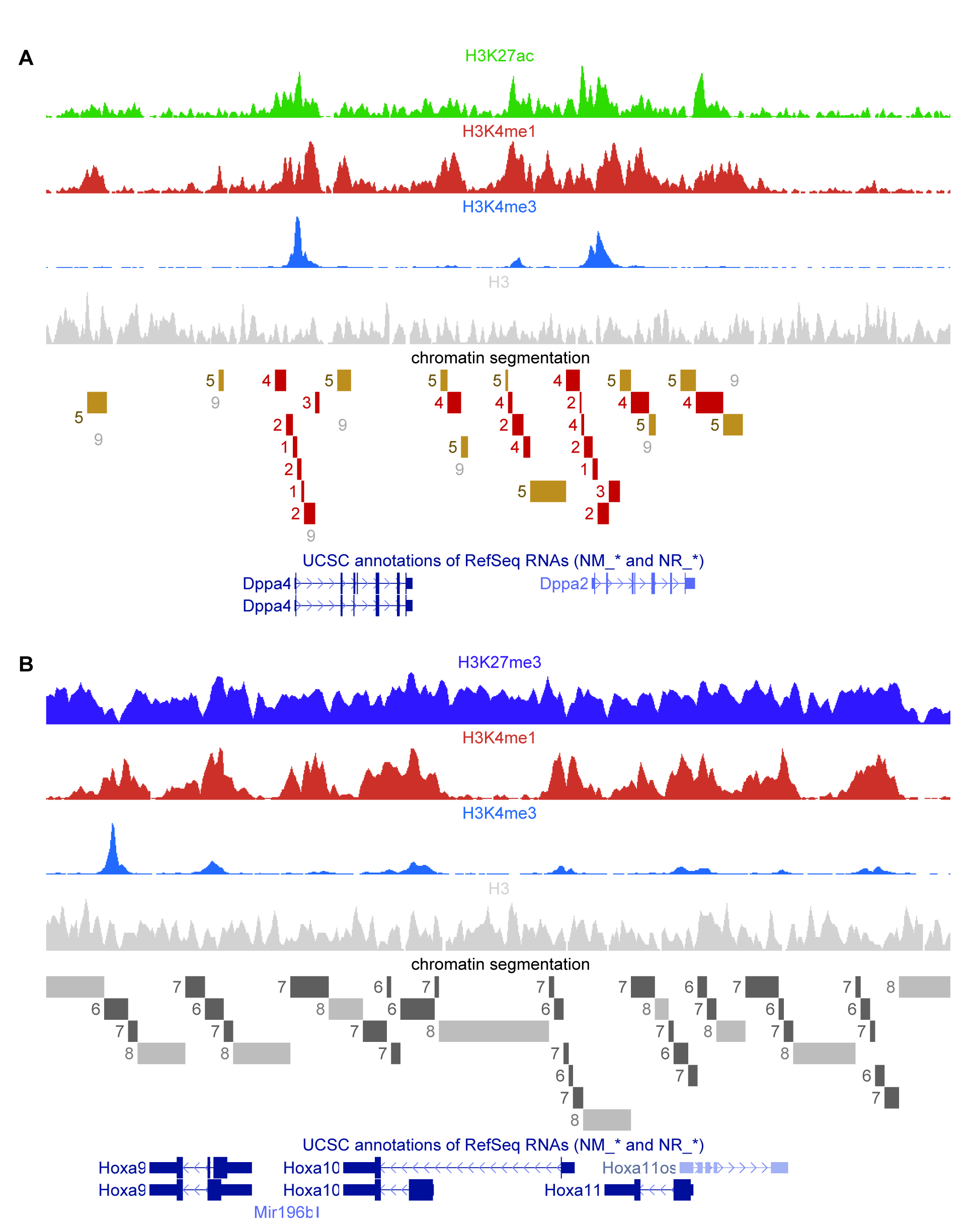

Supplement: S1 Fig — (A) Segments of active states 1–4 cover the same functional regions delimited by peaks of H3K4me3, H3K27ac and H3K4me1. Differences in the definition of active states are due to the shape of the peaks over the same functional elements. The screenshot was taken from the UCSC Genome Browser [62]. (B) Segments of repressed states 6 and 7 denote the sharp peaks of H3K4me3 and H3K4me1 found inside broad regions covered by H3K27me3. State 8 corresponds to the fraction of H3K27me3 peaks that does not overlap with the other two marks. Differences in the definition of repressed states 6 and 7 are due to the shape of the peaks over the same functional elements. The screenshot was taken from the UCSC Genome Browser [62]. (TIF) [file pcbi.1009368.s001.tif]

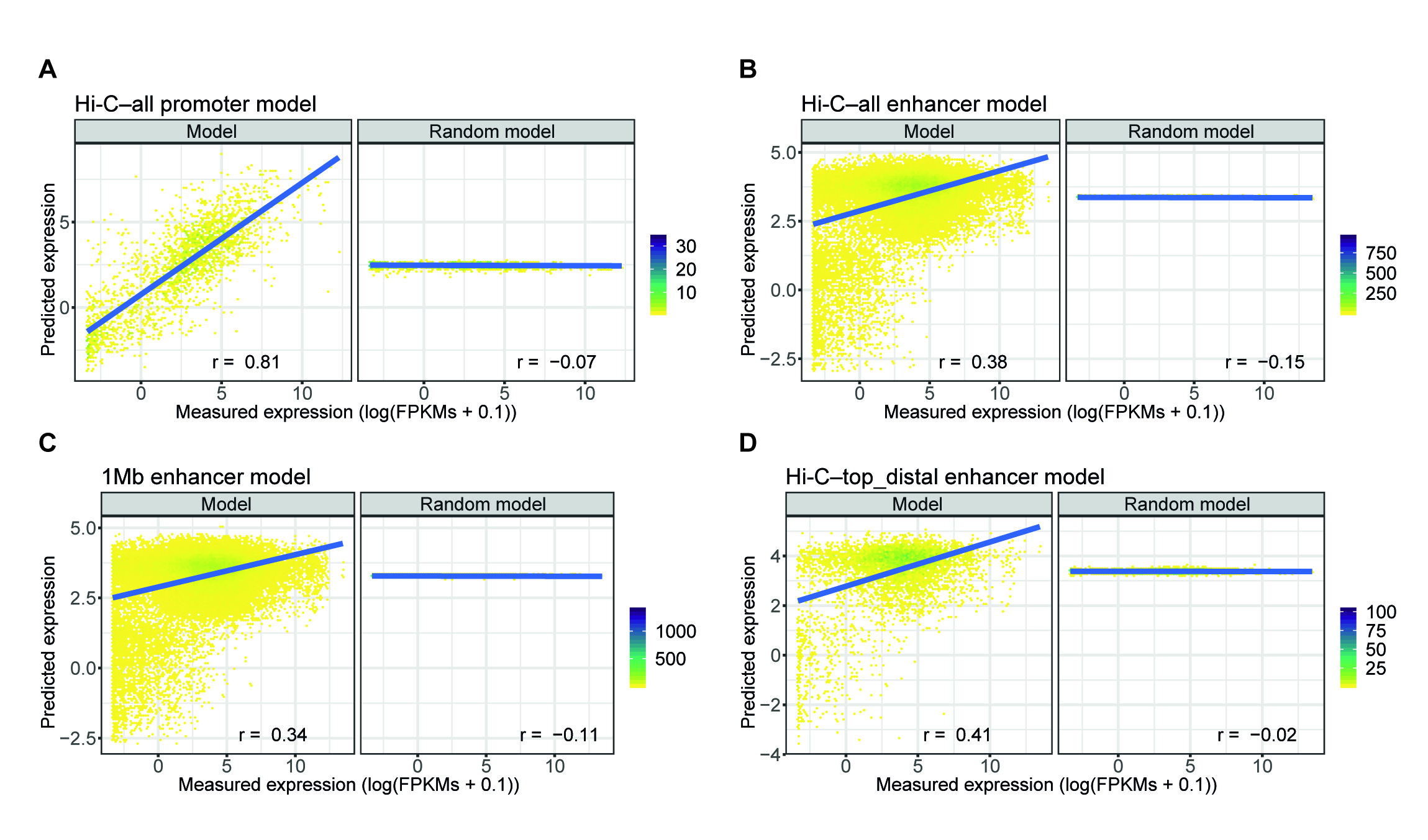

Supplement: S2 Fig — Predicted expression of the test subset of genes calculated by the models versus their measured expression by RNA-seq. Model performances are represented by the Pearson’s correlation (r) between predicted and measured expression values. (A) Left, the model trained on the promoter regions associated to at least one enhancer using all significant interactions of Hi-C (Hi-C–all promoter model). Right, the performance of the same model after randomizing the expression of the training subset of genes. The color bar represents the density of dots. (B) Left, the model trained on the enhancer regions associated to at least one promoter using all the significant interactions of Hi-C (Hi-C–all enhancer model). Right, the performance of the same model after randomizing the expression of the training subset of genes. The color bar represents the density of dots. (C) As for B, but using 1 Mb distance to connect enhancers to promoters. (F) As for B, but using from the Hi-C–top interactions, only distal enhancers (> 5 Kb from a TSS) to generate the model. (TIF) [file pcbi.1009368.s002.tif]

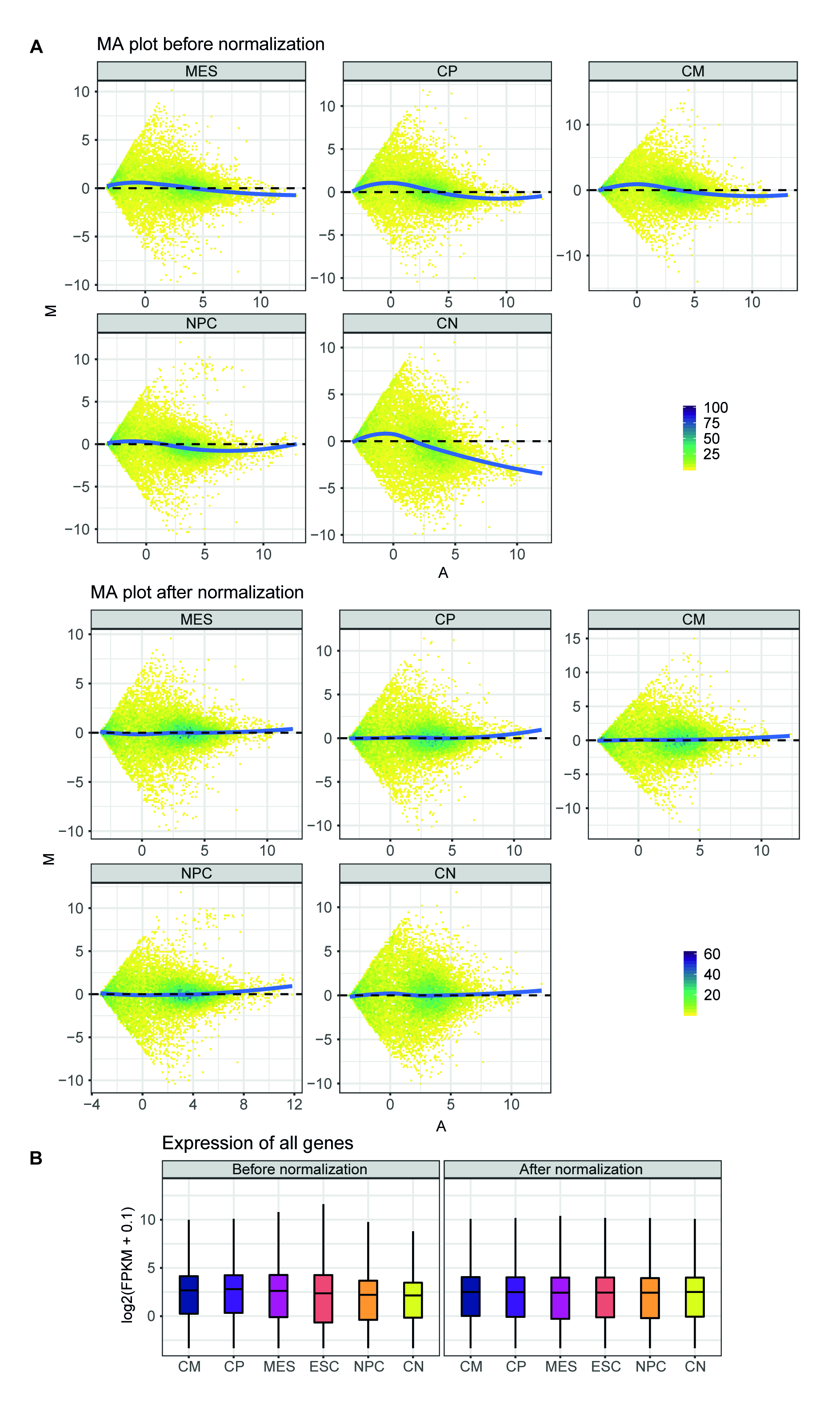

Supplement: S3 Fig — (A) MA plot before and after normalization of expression data at each differentiation time point against ESCs. M represents the log2 ratio of the intensities of the two samples and A is the log2 of the average intensity. Intensity is determined in FPKMs. After normalization, the regression line tends to M = 0. The color bar represents the density of dots. (B) Boxplot of expression of 15,065 protein-coding genes before and after LOESS normalization. CM, cardiomyocytes; CN, cortical neurons; CP, cardio precursors; MES, mesoderm; NPC, neural precursors. (TIF) [file pcbi.1009368.s003.tif]

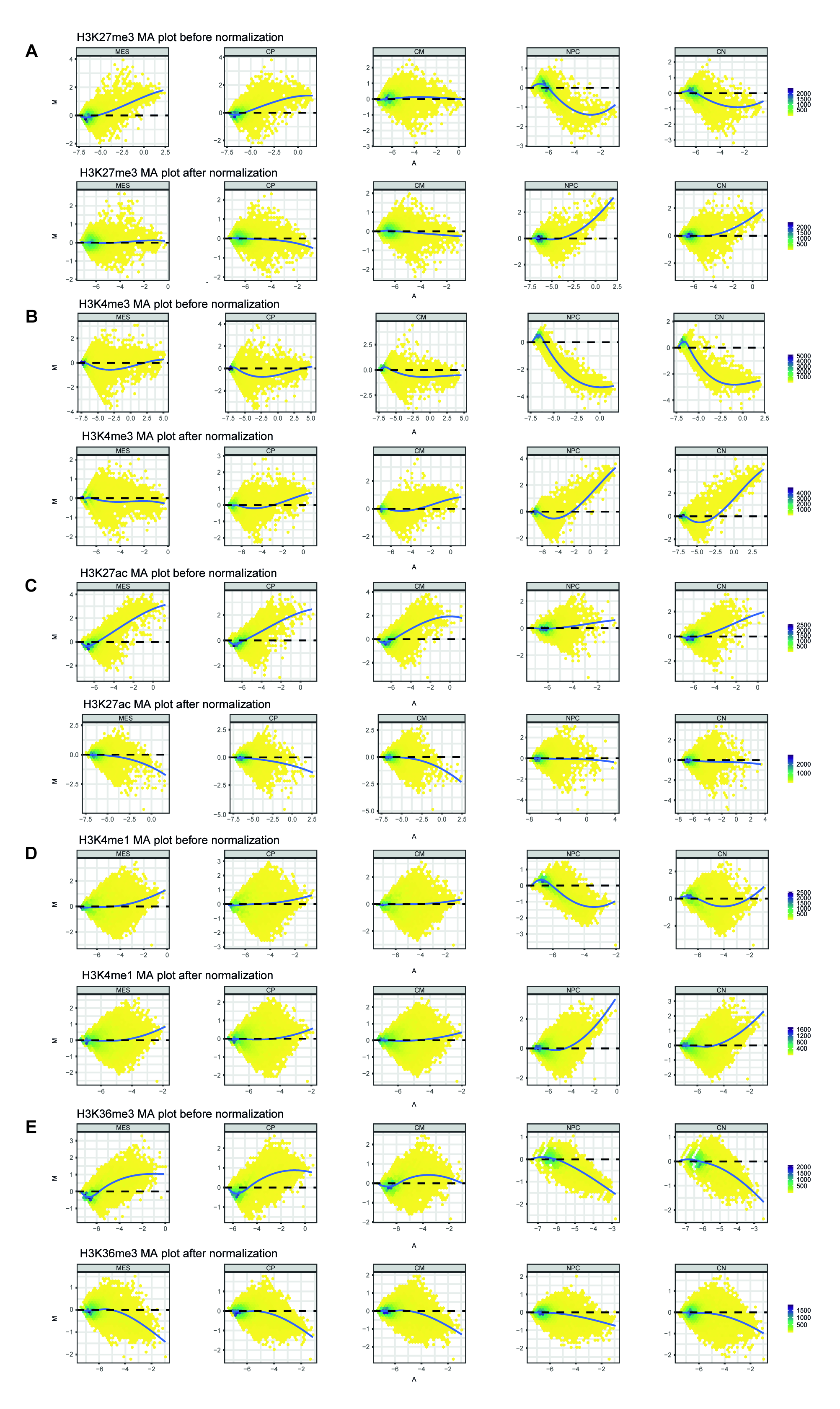

Supplement: S4 Fig — MA plots before and after normalization of each differentiation time point against ESCs. M represents the log2 ratio of the intensities of the two samples, and A is the log2 of the average intensity. Intensity corresponds to normalized count of reads by total number of reads of the ChIP-seq samples of (A) H3K27me3, (B) H3K4me3, (C) H3K27ac, (D) H3K4me1, and (E) H3K36me3. The color bars represent the density of dots. CM, cardiomyocytes; CN, cortical neurons; CP, cardio precursors; MES, mesoderm; NPC, neural precursors. (TIF) [file pcbi.1009368.s004.tif]

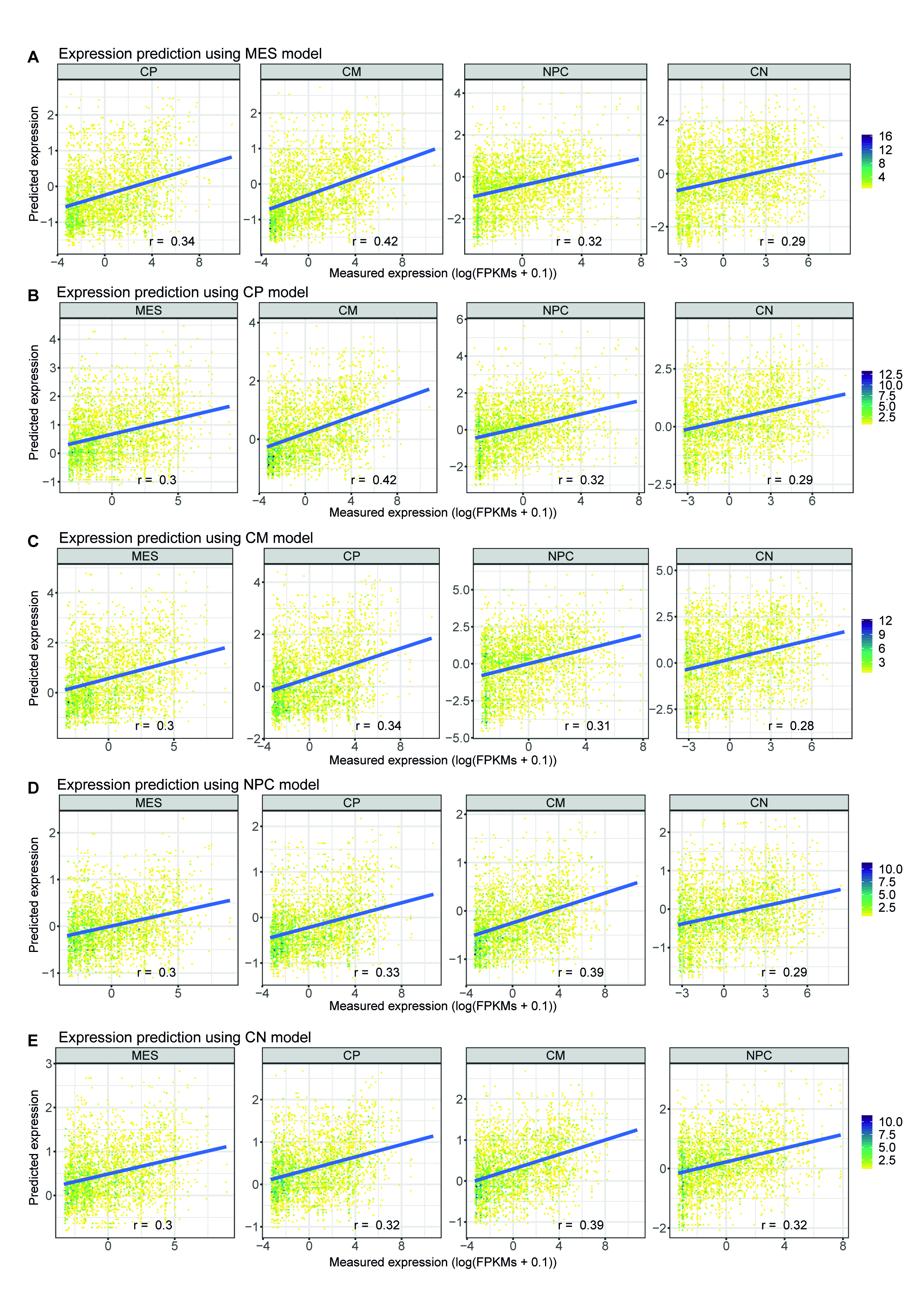

Supplement: S5 Fig — Predicted expression of the test differentiation time points calculated by the models versus their measured expression by RNA-seq. Model performances are represented by the Pearson’s correlation (r) between predicted and measured expression values. The color bars represent the density of dots. (A) Model trained in mesoderm. (B) Model trained in cardio precursors. (C) Model trained in cardiomyocytes. (D) Model trained in neural precursors. (E) Model trained in cortical neurons. CM, cardiomyocytes; CN, cortical neurons; CP, cardio precursors; MES, mesoderm; NPC, neural precursors. (TIF) [file pcbi.1009368.s005.tif]

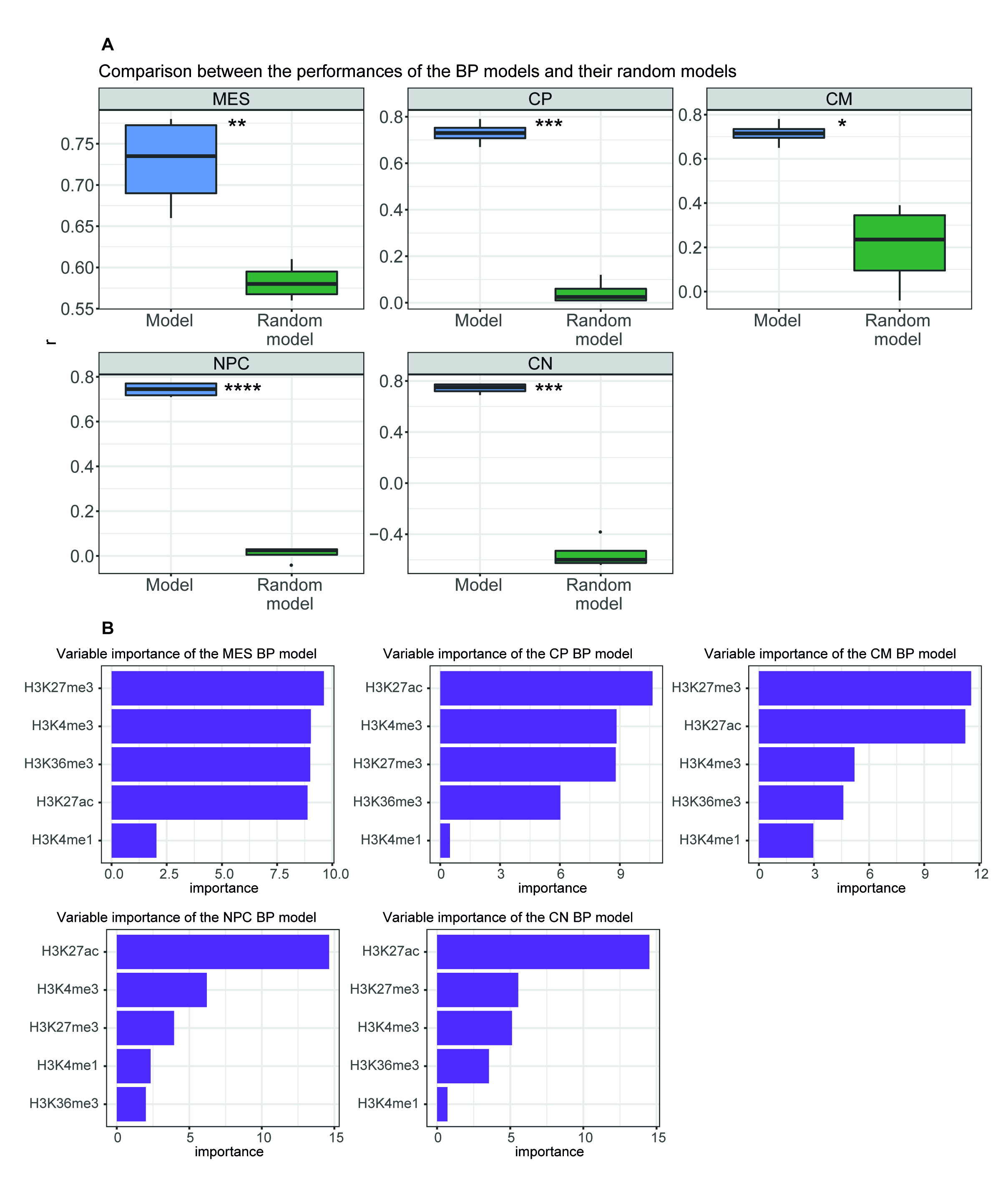

Supplement: S6 Fig — (A) Performance of each differentiation BP model on the rest of the differentiation time points as compared to the performance over the random models. Performance is represented as Pearson’s correlation (r) between predicted expression and measured expression. Significance was assessed using a paired Student’s t-test of the performance of the models or of the random models paired by a differentiation test set (****p < 0.0001, ***p < 0.001, **p < 0.01, *p < 0.05). (B) Importance of histone modifications for each differentiation BP model. Importance is defined as the contribution of each variable in the linear regression predictive model and corresponds to the absolute value of the t-statistics for each model parameter. CM, cardiomyocytes; CN, cortical neurons; CP, cardio precursors; MES, mesoderm; NPC, neural precursors. (TIF) [file pcbi.1009368.s006.tif]

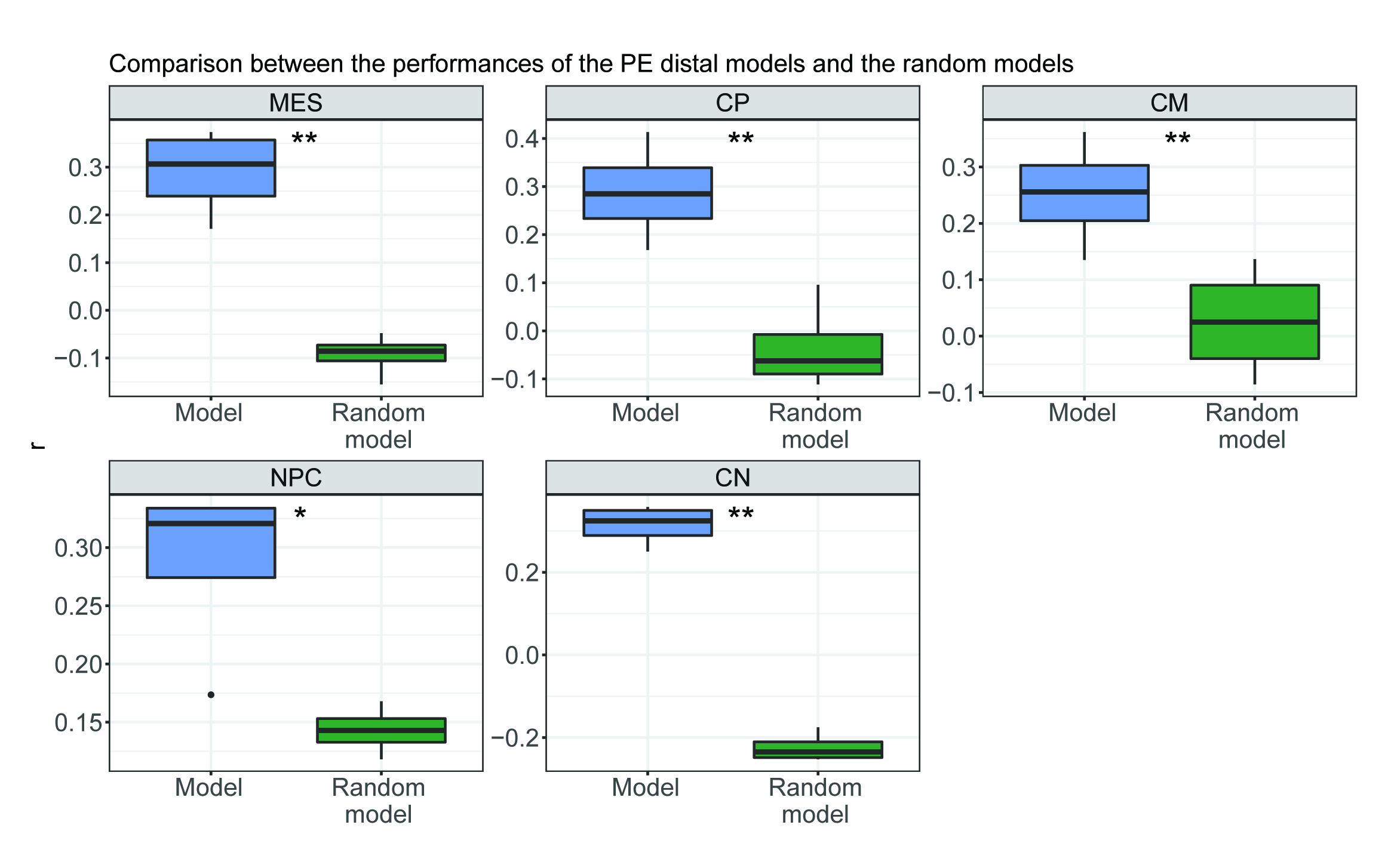

Supplement: S7 Fig — Performance of each differentiation BP model on the rest of the differentiation time points as compared to the performance over the random models. Performance is represented as Pearson’s correlation (r) between predicted expression and measured expression. Significance was assessed using a paired Student’s t-test of the performance of the models or of the random models paired by a differentiation test set (****p < 0.0001, ***p < 0.001, **p < 0.01, *p < 0.05). CM, cardiomyocytes; CN, cortical neurons; CP, cardio precursors; MES, mesoderm; NPC, neural precursors. (TIF) [file pcbi.1009368.s007.tif]

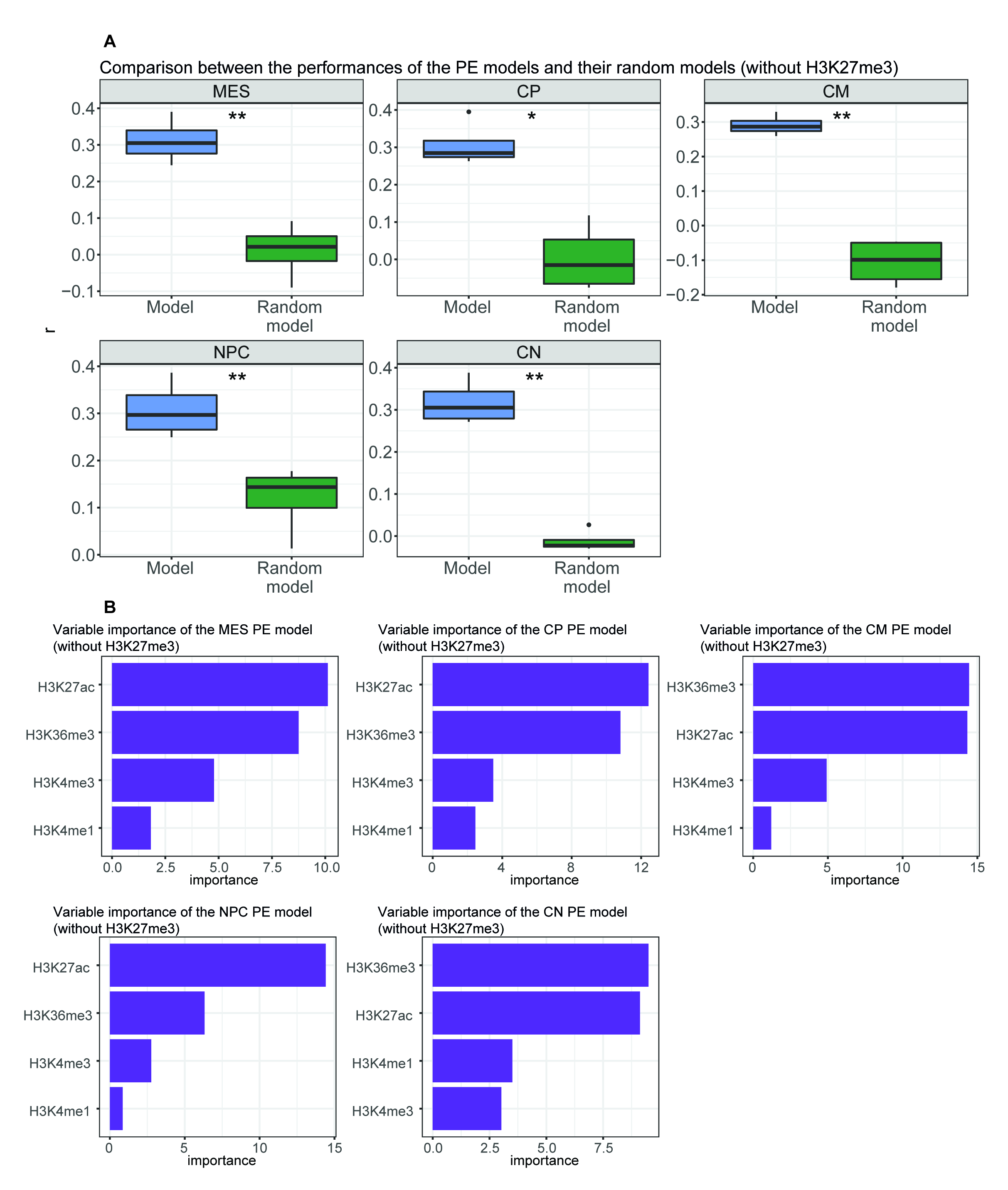

Supplement: S8 Fig — (A) Performance of each differentiation model without H3K27me3 as predictive variable on the rest of the differentiation time points as compared to the performance over the random models. Performance is represented as Pearson’s correlation (r) between predicted expression and measured expression. Significance was assessed using a paired Student’s t-test of the performance of the models or of the random models paired by a differentiation test set (****p < 0.0001, ***p < 0.001, **p < 0.01, *p < 0.05). (B) Importance of histone modifications for each differentiation intragenic model. Importance is defined as the contribution of each variable in the linear regression predictive model and corresponds to the absolute value of the t-statistics for each model parameter. CM, cardiomyocytes; CN, cortical neurons; CP, cardio precursors; MES, mesoderm; NPC, neural precursors. (TIF) [file pcbi.1009368.s008.tif]

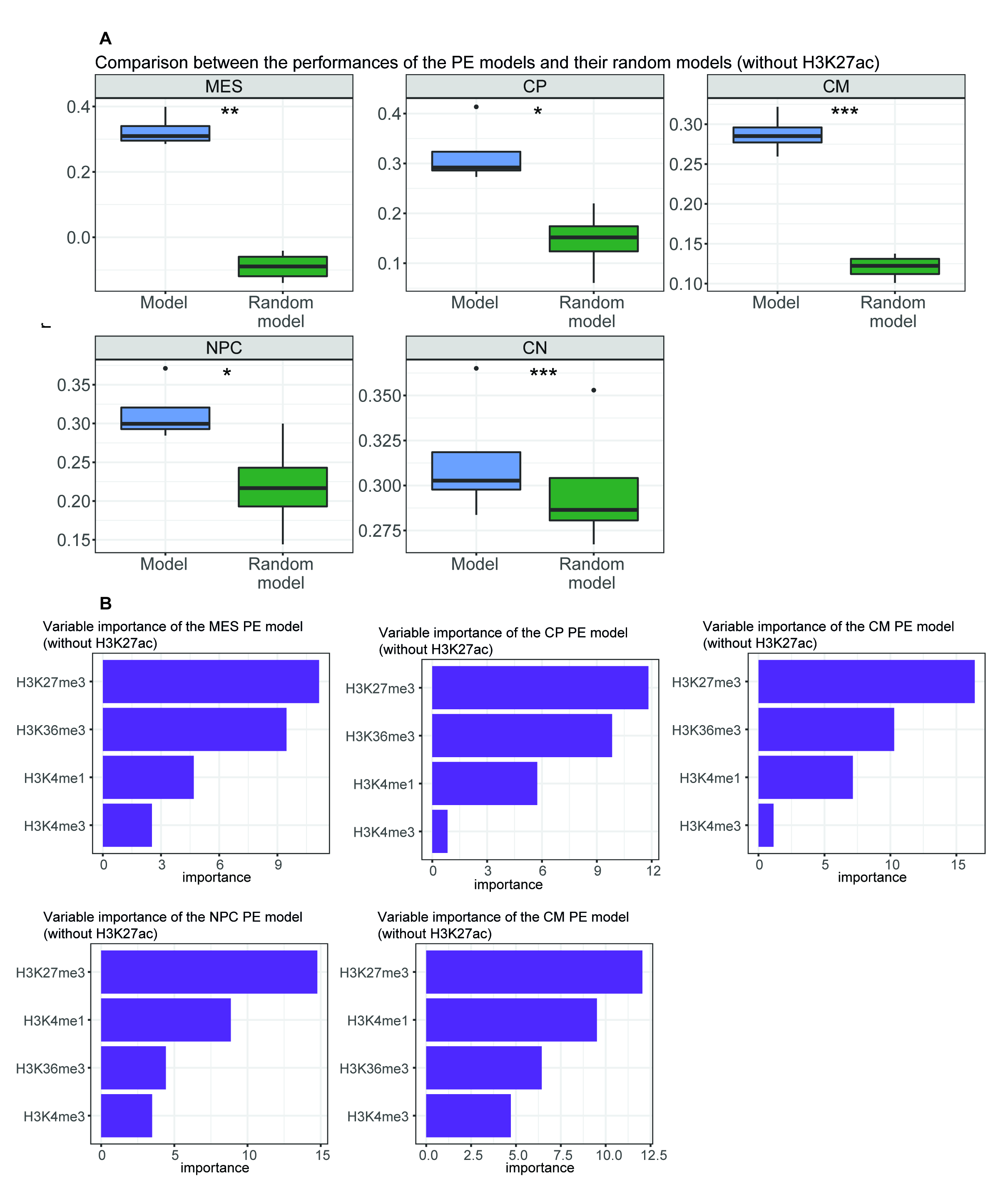

Supplement: S9 Fig — (A) Performance of each differentiation model without H3K27ac as predictive variable on the rest of the differentiation time points as compared to the performance over the random models. Performance is represented as Pearson’s correlation (r) between predicted expression and measured expression. Significance was assessed using a paired Student’s t-test of the performance of the models or of the random models paired by a differentiation test set (****p < 0.0001, ***p < 0.001, **p < 0.01, *p < 0.05). (B) Importance of histone modifications for each differentiation intragenic model. Importance is defined as the contribution of each variable in the linear regression predictive model and corresponds to the absolute value of the t-statistics for each model parameter. CM, cardiomyocytes; CN, cortical neurons; CP, cardio precursors; MES, mesoderm; NPC, neural precursors. (TIF) [file pcbi.1009368.s009.tif]

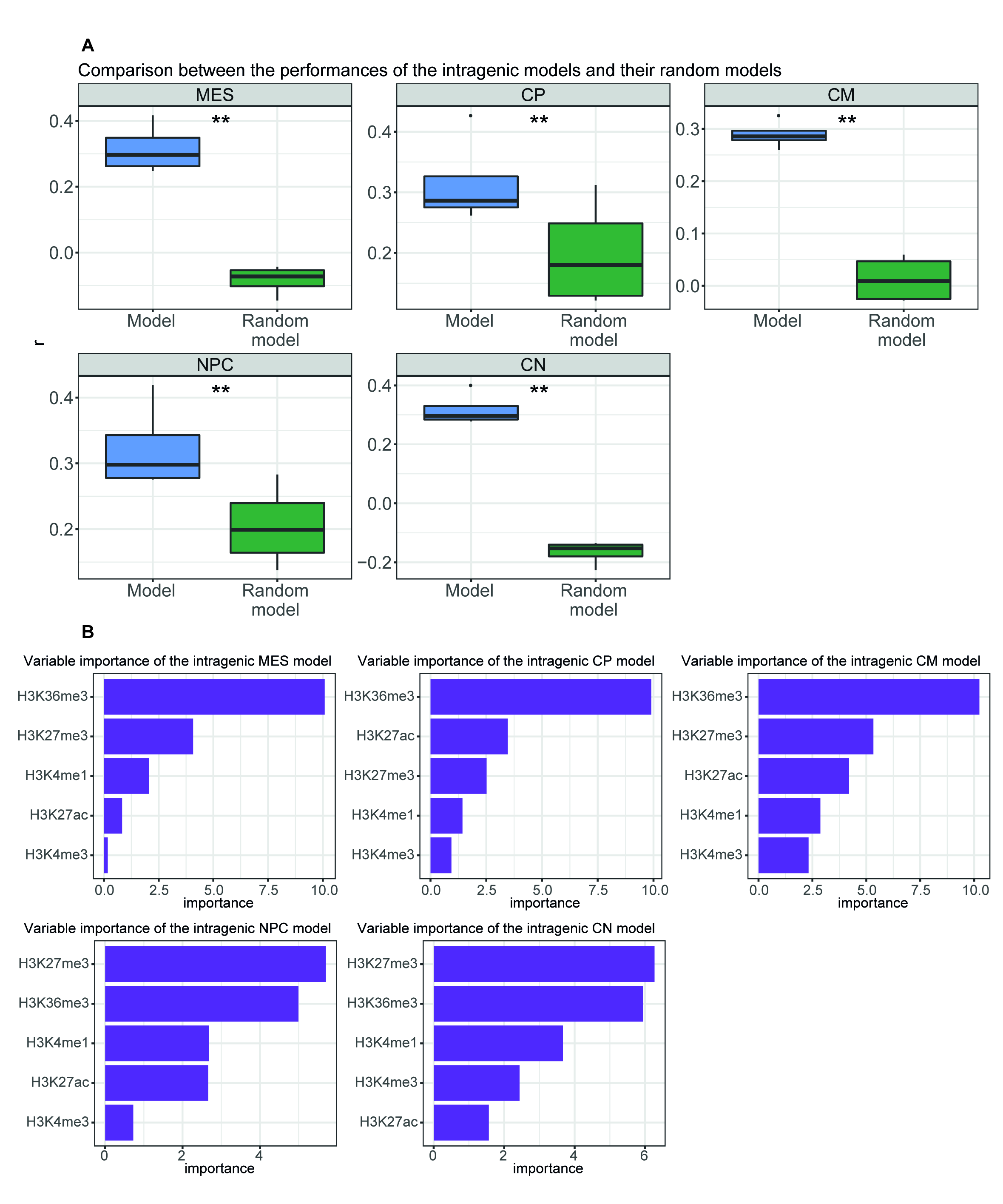

Supplement: S10 Fig — (A) Performance of each differentiation intragenic model on the rest of the differentiation time points as compared to the performance over the random models. Performance is represented as Pearson’s correlation (r) between predicted expression and measured expression. Significance was assessed using a paired Student’s t-test of the performance of the models or of the random models paired by a differentiation test set (****p < 0.0001, ***p < 0.001, **p < 0.01, *p < 0.05). (B) Importance of histone modifications for each differentiation intragenic model. Importance is defined as the contribution of each variable in the linear regression predictive model and corresponds to the absolute value of the t-statistics for each model parameter. CM, cardiomyocytes; CN, cortical neurons; CP, cardio precursors; MES, mesoderm; NPC, neural precursors. (TIF) [file pcbi.1009368.s010.tif]

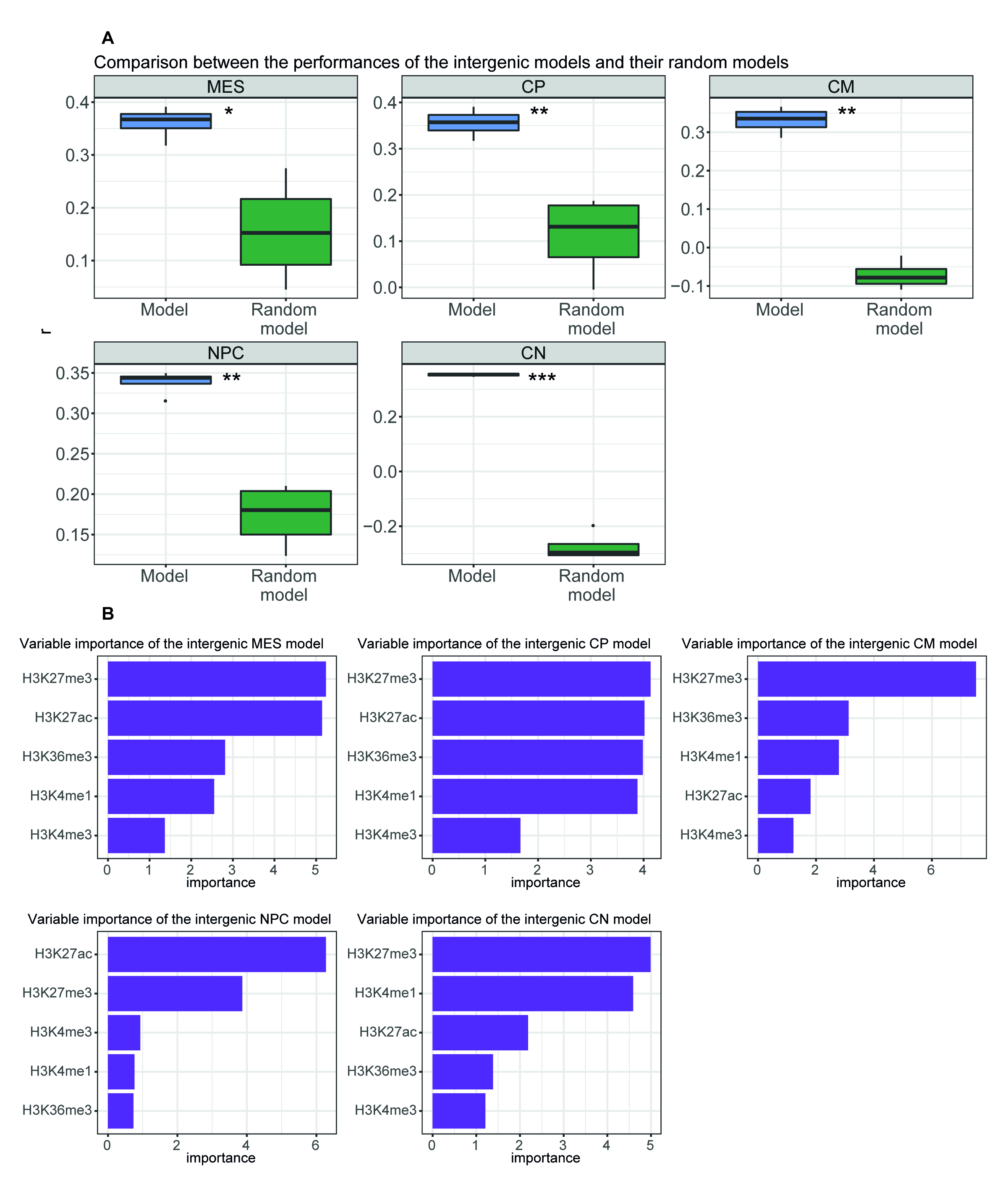

Supplement: S11 Fig — (A) Performance of each differentiation intergenic model on the rest of the differentiation time points as compared to the performance over the random models. Performance is represented as Pearson’s correlation (r) between predicted expression and measured expression. Significance was assessed using a paired Student’s t-test of the performance of the models or of the random models paired by a differentiation test set (****p < 0.0001, ***p < 0.001, **p < 0.01, *p < 0.05). (B) Importance of histone modifications for each differentiation intergenic model. Importance is defined as the contribution of each variable in the linear regression predictive model and corresponds to the absolute value of the t-statistics for each model parameter. CM, cardiomyocytes; CN, cortical neurons; CP, cardio precursors; MES, mesoderm; NPC, neural precursors. (TIF) [file pcbi.1009368.s011.tif]

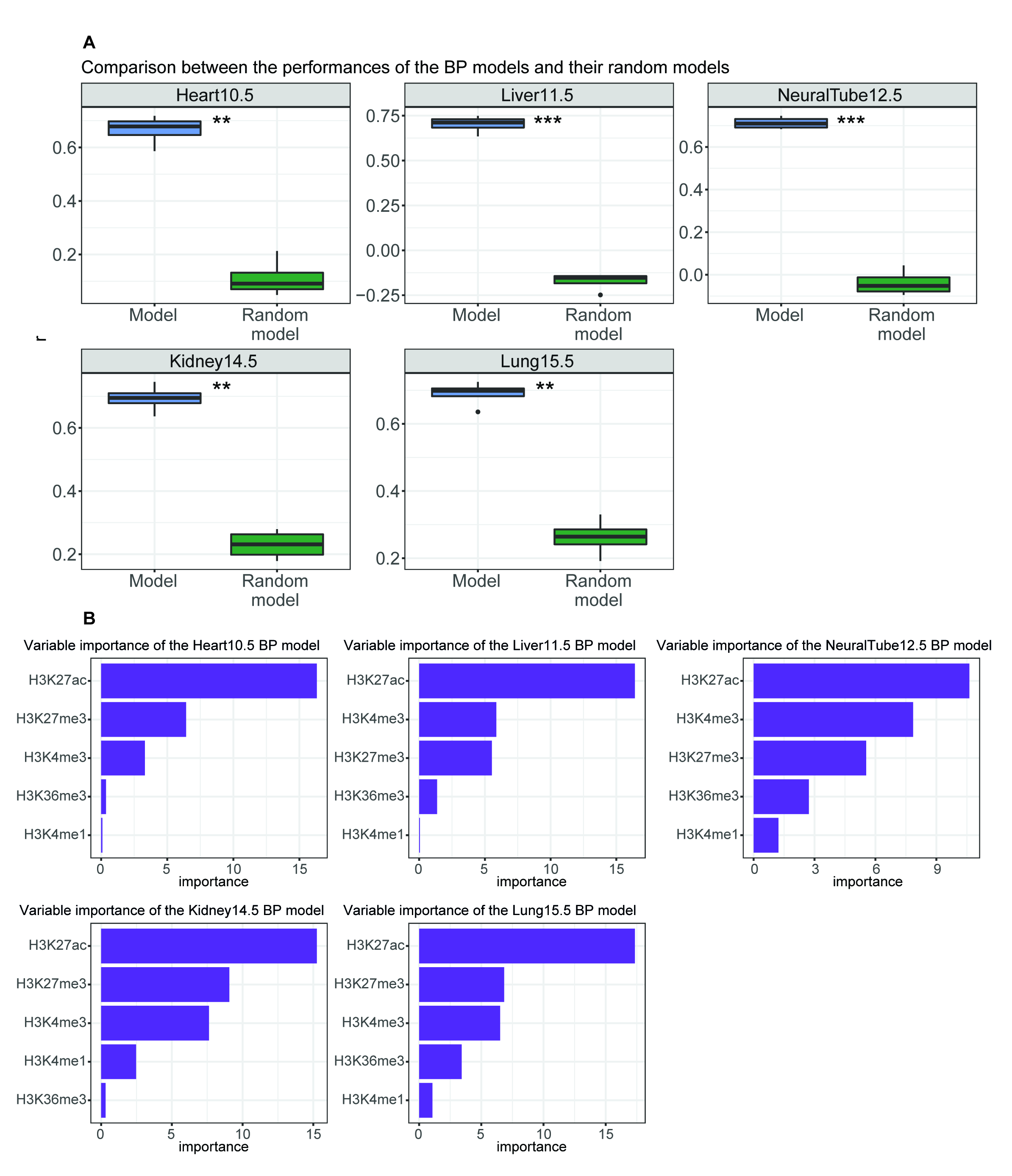

Supplement: S12 Fig — (A) Performance of each differentiation BP model on the rest of the developmental stages as compared to the performance over the random models. Performance is represented as Pearson’s correlation (r) between predicted expression and measured expression. Significance was assessed using a paired Student’s t-test of the performance of the models or of the random models paired by a differentiation test set (****p < 0.0001, ***p < 0.001, **p < 0.01, *p < 0.05). (B) Importance of histone modifications for each development BP model. Importance is defined as the contribution of each variable in the linear regression predictive model and corresponds to the absolute value of the t-statistics for each model parameter. Heart10.5, heart tissue from 10.5 embryonic day; Kidney14.5, kidney tissue from 14.5 embryonic day; Liver11.5, liver tissue from 11.5 embryonic day; Lung15.5, lung tissue from 15.5 embryonic day; NeuralTube12.5, neural tube tissue from 12.5 embryonic day. (TIF) [file pcbi.1009368.s012.tif]
